# Two-dimensional perovskites with alternating cations in the interlayer space for stable light-emitting diodes

**DOI:** 10.1515/nanoph-2021-0037

**Published:** 2021-04-29

**Authors:** Yiyue Zhang, Masoumeh Keshavarz, Elke Debroye, Eduard Fron, Miriam Candelaria Rodríguez González, Denys Naumenko, Heinz Amenitsch, Joris Van de Vondel, Steven De Feyter, Paul Heremans, Maarten B. J. Roeffaers, Weiming Qiu, Bapi Pradhan, Johan Hofkens

**Affiliations:** Molecular Imaging and Photonics, Department of Chemistry, KU Leuven, Celestijnenlaan 200F, Leuven, 3001, Belgium; Institute of Inorganic Chemistry, Graz University of Technology, Stremayrgasse 9/V, Graz, 8010, Austria; Quantum Solid-State Physics (QSP), Department of Physics and Astronomy, KU Leuven, Celestijnenlaan 200D, Leuven, 3001, Belgium; Imec, Kapeldreef 75, Leuven, 3001, Belgium; cMACS, Department of Microbial and Molecular Systems, KU Leuven, Celestijnenlaan 200F, Leuven, 3001, Belgium; Max-Planck-Institute for Polymer Research, 55128 Mainz, Germany; ESAT, KU Leuven, Kasteelpark Arenberg 10, Leuven, 3001, Belgium

**Keywords:** alternating interlayer cations, light-emitting diodes, stability, two-dimensional perovskites

## Abstract

Lead halide perovskites have attracted tremendous attention in photovoltaics due to their impressive optoelectronic properties. However, the poor stability of perovskite-based devices remains a bottleneck for further commercial development. Two-dimensional perovskites have great potential in optoelectronic devices, as they are much more stable than their three-dimensional counterparts and rapidly catching up in performance. Herein, we demonstrate high-quality two-dimensional novel perovskite thin films with alternating cations in the interlayer space. This innovative perovskite provides highly stable semiconductor thin films for efficient near-infrared light-emitting diodes (LEDs). Highly efficient LEDs with tunable emission wavelengths from 680 to 770 nm along with excellent operational stability are demonstrated by varying the thickness of the interlayer spacer cation. Furthermore, the best-performing device exhibits an external quantum efficiency of 3.4% at a high current density (J) of 249 mA/cm^2^ and remains above 2.5% for a J up to 720 mA cm^−2^, leading to a high radiance of 77.5 W/Sr m^2^ when driven at 6 V. The same device also shows impressive operational stability, retaining almost 80% of its initial performance after operating at 20 mA/cm^2^ for 350 min. This work provides fundamental evidence that this novel alternating interlayer cation 2D perovskite can be a promising and stable photonic emitter.

## 1 Introduction

Two-dimensional (2D) metal halide perovskites have recently attracted broad attention due to their impressive optoelectronic properties for various applications [1], [2], [3], such as solar cells [4, 5], photodetectors [6] and light-emitting diodes (LEDs) [7], [8], [9]. To date, 2Dlayered Ruddlesden-Popper (RP) phase perovskites are the most common structural type that has been intensively studied. Generally, they have demonstrated superior stability against humidity compared to 3D perovskites [10], [11], [12] since the long organic spacer cations in RP perovskites make them more hydrophobic, preventing moisture induced degradation. Furthermore, the high dielectric contrast between the inorganic slab and the organic spacer hence, the presence of a dielectric quantum confinement results in a much larger exciton binding energy compared to 3D perovskites [7], leading to enhanced radiative recombination that is beneficial for LED applications. Indeed, perovskite LEDs (PeLEDs) based on RP perovskites have been demonstrated to have high external quantum efficiencies (EQEs) over 20% in recent years [13], [14], [15], [16], [17]. The operational stability of these devices, however, is unfortunately far from satisfactory, with half-lives ranging from only a few minutes to a few hours depending on the electrical bias. In addition, the long chain spacer cation has been proven to severely hamper charge transport within the device [4, 18]. Therefore, a high driving voltage is required which is unfavorable for prolonged device operational stability. Another type of 2D perovskite, i.e., the Dion-Jacobson (DJ) structure, was introduced by Ning et al. [19] and has been used in PeLEDs. Due to the higher binding energy between the layered structures and higher molecular dissociation energy of the DJ structure than that of the RP structure, a significant improvement in operational stability was observed for the devices using the DJ structure. This indicates that the chemical and crystal nature of 2D perovskites play a crucial role in determining the performance of PeLEDs.

To improve the structural properties of 2D perovskites even further, a novel approach with two different alternating cations in the interlayer space (ACI) has been introduced. ACI perovskites using guanidinium (GA) and methylammonium (MA) as the alternating spacer cations were first reported by Kanatzidis et al. [20]. The ACI perovskites have an empirical chemical formula of (GA)(MA)_n_Pb_n_I_3n+1_. However, the unique ordering of the spacer cations makes their crystal structure reminiscent of that of DJ perovskites with a relative (1/2, 0) shift of the layers along the ab-plane, though the occupation of the interlayer sites is different due to charge balance restrictions [20]. Such a unique structure of ACI perovskites could lead to different photophysical and optoelectronic properties compared to the known 2D perovskites, especially considering that the replacement of the large cations with two different smaller cations will result in a much closer packing of the [Pb_n_I_3n+1_] slabs [20, 21]. Recently, by controlling the processing conditions of ACI perovskites, solar cells with power conversion efficiencies over 18% have been fabricated, demonstrating the potential of such 2D perovskites for high-performance photonic devices [21], [22], [23]. Nevertheless, to the best of our knowledge, the employment of ACI perovskites in PeLEDs has not been evaluated despite the beneficial properties that can be expected, e.g., (1) improved charge transport due to the use of smaller spacer cations and (2) an increased number of hydrogen bonds and consequently a stronger crystal lattice due to the use of GA cations [24], [25], [26], [27].

Herein, we present the first demonstration of PeLEDs based on ACI perovskites. Various characterizations were first applied to confirm the formation of ACI perovskites and to investigate their film morphology, crystal structure, optical properties, and photoluminescence temperature evolution. In particular, synchrotron-based grazing incidence wide angle X-ray scattering (GIWAXS) measurements of thin films confirm the phase purity of the ACI perovskites. In addition, transient absorption spectroscopy (TAS) was used to obtain insight into the charge carrier dynamics [28], [29], [30] in the ACI perovskites prepared with different *n* values, showing efficient charge funneling for the ACI perovskite with *n* = 3. Therefore, PeLEDs with a maximum EQE of 3.4% were achieved by using the ACI perovskite with *n* = 3 in a typical LED structure of ITO/PolyTPD/perovskites/TPBi/LiF/Al, where PolyTPD and TPBi refer to poly[N,N′-bis(4-butylphenyl)-N,N′-bis(phenyl)-benzidine] and 2,2′,2″-(1,3,5-benzinetriyl)-tris(1-phenyl-1-H-benzimidazole), respectively [8, 31, 32]. Devices based on ACI retain approximately 80% of their initial performance after operating at 20 mA/cm^2^ for 350 min, outperforming the operational stability of PeLEDs fabricated with the same device structure but different perovskites [8, 17, 33]. This demonstrates the initial promise of ACI perovskites for stable PeLED application.

## 2 Results and discussion

### 2.1 Crystal structure, morphology, and optical properties of ACI perovskite thin films

**Figure 1:**
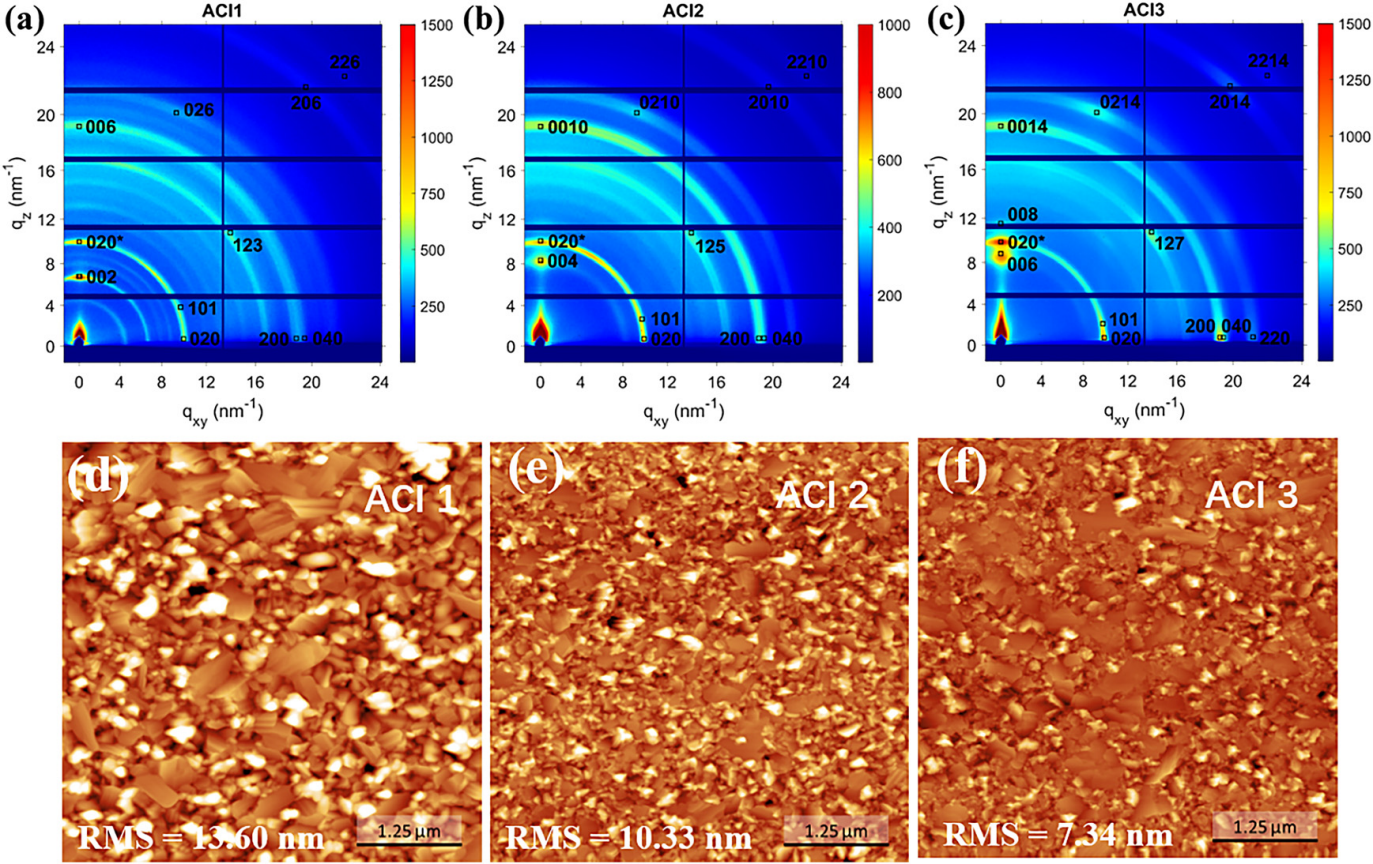
(a–c) 2D GIWAXS patterns of (GA)(MA)_n_Pb_n_I_3n+1_ (ACI, *n* = 1–3) films acquired at the incidence angle of 0.5°.The reflections are indexed according to the adjusted unit cell parameters given in Table S1, (d–f) AFM images (5 × 5 μm) and RMS roughness of the ACI films (roughness with standard deviation, ACI 1: 13.60 ± 5.53 nm, ACI 2: 10.33 ± 2.46 nm, ACI 3: 7.34 ± 1.98 nm).

ACI perovskite films, i.e., (GA)(MA)_n_Pb_n_I_3n+1_ with *n* = 1, 2, and 3 layers (denoted ACI 1, ACI 2 ,and ACI 3), were fabricated using a one-step antisolvent method of a precursor solution with the dripping of toluene during spincoating. The as-cast films were then annealed at a low temperature (70 °C) for 5 min to form perovskite films [21, 31]. We first studied the crystal structure of the obtained ACI perovskite films using grazing-incidence X-ray diffraction (GIWAXS) measurements (Figure 1(a–c)) that have been extensively used in recent years to characterize 2D and 3D perovskites [20, 21, 23, 34], [35], [36], [37]. All ACI films show diffuse diffraction rings with strong diffraction spots indicating a preferential orientation of the perovskite grains with respect to the substrate surface [38]. IIn order to determine lattice parameters of organized nanostructures in thin films and to investigate the crystal orientation relative to the substrate, three-dimensional structure indexing has been performed and overlayed [39]. Using the data obtained on single ACI (*n* = 1–3) crystals [20], the powder diffraction patterns for corresponding crystalline phases have been simulated and compared to the azimuthal integration of GIWAXS data of ACI films (Figure S1). All peaks which exceed 30% of maximum simulated intensity value in the powder diffraction have been identified in the GIWAXS patterns. The lattice parameters have been adjusted (Table S1) to match the prominent reflections in GIWAXS [40] (Figure 1(a–c)). A 5% increase in the lattice parameter along c of axis is observed for all ACI films compared to corresponding single crystal data [20]. The scattering features located at *q* = 4.5 nm^−1^ and around 8 nm^−1^ for the ACI 1 film (Figure 1(a)) suggest the presence of an intermediate MAPbI_3_ perovskite phase [21]. The ACI 3 film (Figure 1(c)) exhibits more pronounced in-plane and out-of-plane Bragg spots suggesting higher crystalline order in which crystal domains are preferentially oriented along their *b* and *c* axes perpendicular to the substrate. In order to quantify the degree of domain orientation with respect to the substrate, the azimuthal distributions of the reflections from (002), (004), and (006) planes (ACI 1–3 films, respectively) have been analyzed (Figure S2). Referring to the (00l) planes, the Hermans’ orientation function <*P*(*θ*)> has been calculated which provides an alternative interpretation of crystal orientation in relative to the interpretation of the azimuthal distribution of (020/101) peak as proposed in literature [21, 34]. <*P*(*θ*)> can vary between −0.5 and 1. When all the crystals in the film are oriented with their c axes parallel to the out-of-plane direction (the reference direction in our case) <*P*(*θ*)> is 1, when all the *c* axes are perpendicular to the reference direction (i.e. in-plane direction) <*P*(*θ*)> is −0.5, and when the crystals are randomly oriented <*P*(*θ*)> is 0. As it can be seen from Figure S2(b) the highest degree of orientation is observed for ACI 3 film (<*P*(*θ*)> = 0.28), while ACI 2 film has a very poor orientation (<*P*(*θ*)> = 0.06), and ACI 1 film shows an intermediate degree of orientation (<*P*(*θ*)> = 0.14).

The morphology of the ACI perovskite films was characterized by atomic force microscopy (AFM) measurements (Figure 1(d–f)). The AFM images show that for all three samples, the crystal grains are close-packed, but the topography and surface roughness are different. More detailed topography is shown in Figure S3. Many high features can be observed in the ACI 1 films, indicating a rougher surface with a root-mean-square (RMS) roughness of 13.60 nm. The number of such high features decreases with increasing *n*, as does the surface roughness, i.e., 10.33 and 7.40 nm for ACI 2 and ACI 3, respectively.

**Figure 2:**
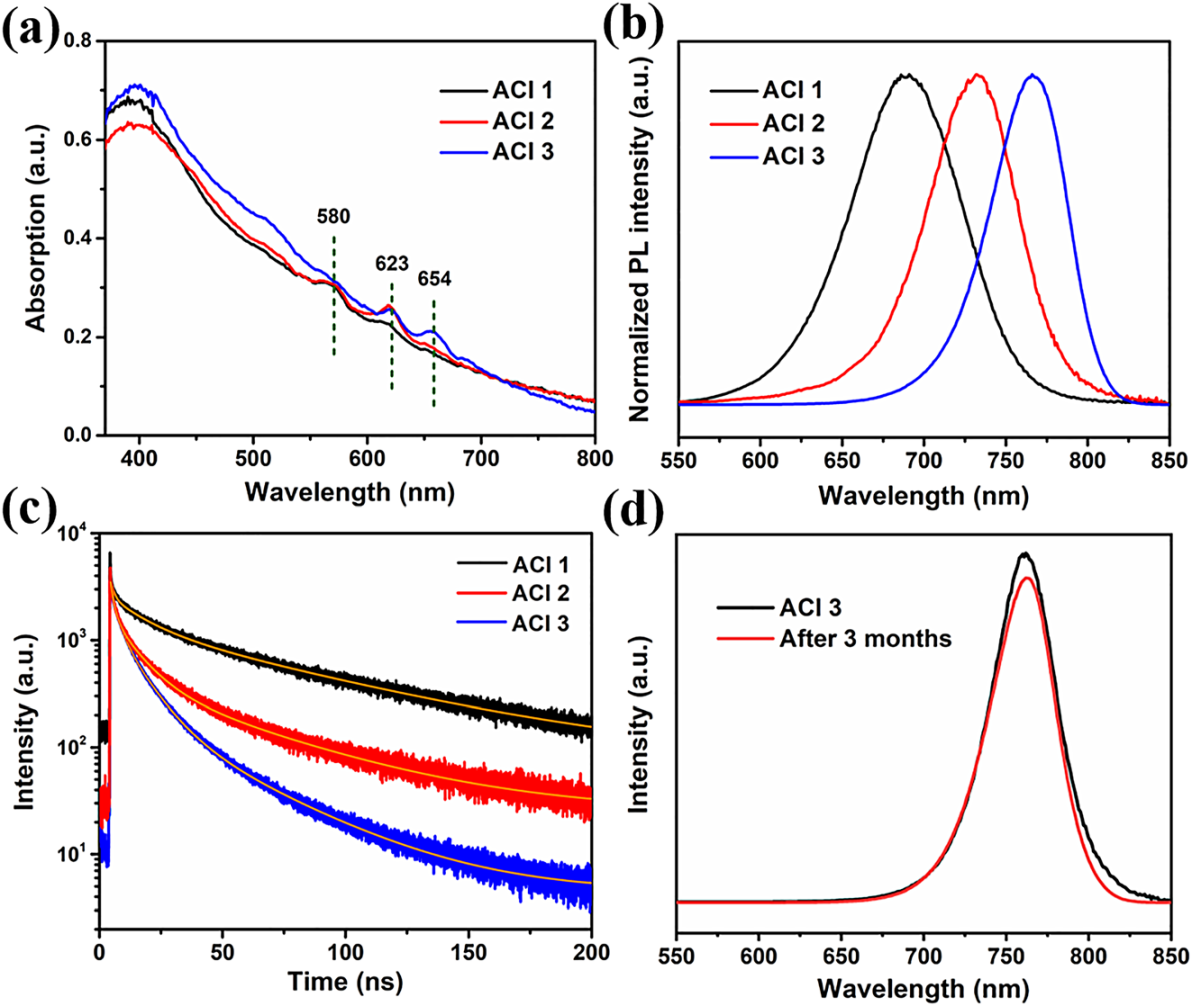
(a) UV–vis absorption spectra, (b) steady-state PL spectra excited at 470 nm, (c) TRPL spectra of the ACI (*n* = 1, 2, 3) perovskite films, and (d) PL spectra of ACI 3 films measured at Day 0 and Day 90 when stored in air.

Figure 2(a) shows the UV–vis absorption spectra of the ACI perovskite (GA)(MA)_n_PbnI_3n+1_ films with different *n* values. All three thin films exhibit a band edge absorption position close to that of the MAPbI_3_ bulk phase, which suggests the existence of the bulk phase [41]. The absorption intensity of the ACI films in the range of ≈400–800 nm slightly changes with the GA/MA ratio. Similar to RP perovskites, the intensity of the excitonic peaks decreases with decreasing GA/MA ratio in the ACI perovskites as a consequence of the decrease in the dielectric confinement [20, 33]. Beyond the fundamental exciton *n* = 1 at 1.9 eV (654 nm), where *n* is an integer, we also observed harmonics with integer *n* equal to 2 and 3 at 2 eV (623 nm), and 2.14 eV (580 nm), representing different quantum well (QWs) structures (detailed in Figure S4) [20, 22]. In addition to the excitonic peaks, the ACI films simultaneously exhibit other high-energy peaks as weak shoulders from the preceding lower-dimensional perovskites, suggesting that the ACI films are a mixture of perovskite QWs with different bandgaps [42]. The absorption edge and the excitonic peak position are redshifted in the ACI perovskites compared to RP perovskites. This is also reflected in the color of the (GA)(MA)_n_PbnI_3n+1_ compound, which changes from orange-red (*n* = 1) to brown (*n* = 2) to dark brown (*n* = 3) (Figure S5). According to the Kubelka-Munk theory, the band edges can be calculated as 2.05, 1.99, and 1.84 eV for the three QWs (Figure S6), suggesting the influence of the different GA/MA ratios, i.e., perovskite dimensionality tuning, on the band structure of the materials [20].

For ACI 1, ACI 2, and ACI 3 photoluminescence (PL), the maximum emission peaks are located at 680, 740, and 770 nm, respectively (Figure 2(b)). Notably, the FWHM of the ACI 1 peak is greater than that of ACI 2 and ACI 3, attributed to the existence of multiple emitting species. A complete overview of the various excitation wavelengths and emitting species of all systems under study is represented in Figure S7 through their excitation-emission maps. The PL homogeneity of the ACI samples can be directly observed in Figure S7. Note that further increasing the GA concentration beyond that of ACI 1, without dimethyl sulfoxide (DMSO) in the precursor, and inappropriate addition of antisolvent can cause phase separation, see Figure S8 [28]. Herein, the crystal growth and orientation of the 2D perovskites could be induced by the strong coordination molecule DMSO when it was used in the precursor solution, leading to a decreased trap state density and more efficient charge transport compared to the normal 2D perovskite film [36]. The carrier lifetimes of the different ACI films were studied by time-resolved PL (TRPL) measurements (Figure 2(c)). The luminescence lifetimes of ACI 1, ACI 2, and ACI 3 films are compiled in Table S2. The shortest lifetime was recorded for the ACI 3 film, indicating the presence of faster radiance recombination. Note that PL linewidth broadening has a direct consequence with carrier phonon interactions. Strong carrier-phonon interaction has been invoked to account for below-band-gap self-trap of exciton states and broad emissions in quasi-2D layer perovskites. Self-trapping of exciton becomes more prominent with a decreasing number of *n*, since the reduction of the dimensionality of a crystalline system lowers the deformation energy, facilitating self-trapping [43], [44], [45]. Therefore, we attribute that the ACI 1 contains more phonon assisted exciton recombination in addition to free exciton recombination which increases the average lifetime of ACI 1 [40]. Finally, the environmental stability of the optimized ACI 3 film was evaluated (Figure 2(d)), showing a marginal PL intensity change after being stored in air for 3 months in the dark as expected for such systems.

**Figure 3:**
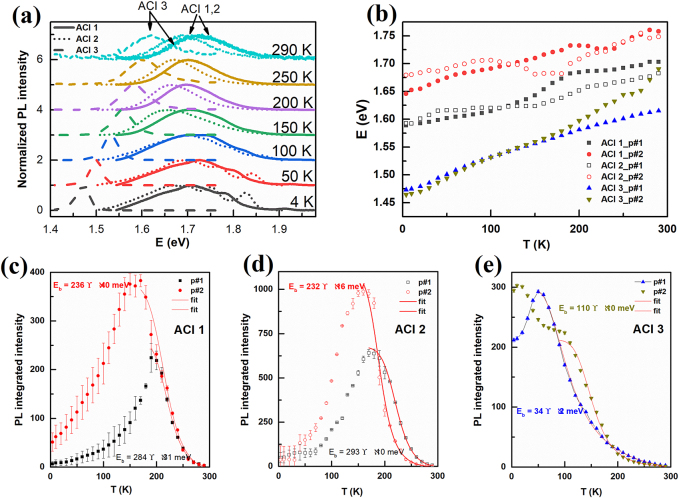
Temperature evolution of the PL properties of ACI 1, 2, and 3. (a) PL spectra at different temperatures for ACI 1, 2, and 3. The two peaks (annotated as p#1 and p#2) indicated by arrows for *n* = 1, 2 and for *n* = 3 are considered for further analysis, (b) temperature dependence of the PL peak centers of the peaks indicated by arrows in (a) for all three materials, (c–e) PL integrated intensity as a function of temperature (*T*) for ACI 1 (c), ACI 2 (d) and ACI 3 (e). The exciton binding energies are extracted using the Arrhenius equation shown by solid lines in Figure (c–e).

To obtain deeper insight into the photophysical properties of the systems under study, we investigated the temperature evolution of their PL between 300 and 4.2 K. PL spectra have been collected at an increment of 5 K for all materials. PL spectra at selected temperatures are depicted in Figure 3(a) (PL spectra for all temperatures are shown in Figure S9). For ACI 1 and 2, several PL peaks are observed at lower temperatures whose origin is under debate and has been attributed to the presence of multiexciton correlations (many-body electron-hole correlations), free and bound excitons as well as bi‐excitons or coexistence of different phases of 2D perovskites [46]. ACI 3 however, shows two peaks down to 4.2 K. Note that for all three systems, we considered the two peaks present at room temperature for further analysis (shown by arrows in Figure 3(a) and annotated as p#1 and p#2 in Figure 3(b–e)). An example of the fitting procedure and the two Gaussian peaks extracted for all materials at room temperature is depicted in Figure S10. The temperature dependence of the two PL peak centers is depicted in Figure 3(b), exhibiting approximately the same behavior for all three systems, with no abrupt changes and hence no structural phase transitions.

The exciton binding energy of all systems under study was estimated by applying the Arrhenius equation to the temperature dependence of their PL integrated intensity, as illustrated in Figure 3(c–e):

$$J\left(T\right)=I_{0}/\left(1+Ae^{-E_{b}/k}B^{T}\right)$$

where *I*_0_ and A are constants, kB is the Boltzmann constant, and Eb is the exciton binding energy. The solid curves are the fits. The exciton binding energy for ACI 3 decreases drastically (Eb = 110 meV) compared to those of ACI 1 and ACI 2 (on the order of 200–300 meV) due to the reduced quantum confinement effect in ACI 3 [28, 47]. It is noteworthy that the exciton binding energy of ACI 3 is still much larger than that of common MAPbI_3_ perovskites [48, 49]. Observing such a low exciton binding energy for ACI 3 hints at a transition from a confined 2D system (i.e., ACI 1 and 2) to a bulk system when varying the number of layers in the structure. Furthermore, the fact that more peaks are observed for ACI 1 and 2 at lower temperatures points towards their more confined structures where many-body electron-hole correlations are more prominent compared to ACI 3 [46].

### 2.2 TAS study on the obtained ACI perovskites

**Figure 4:**
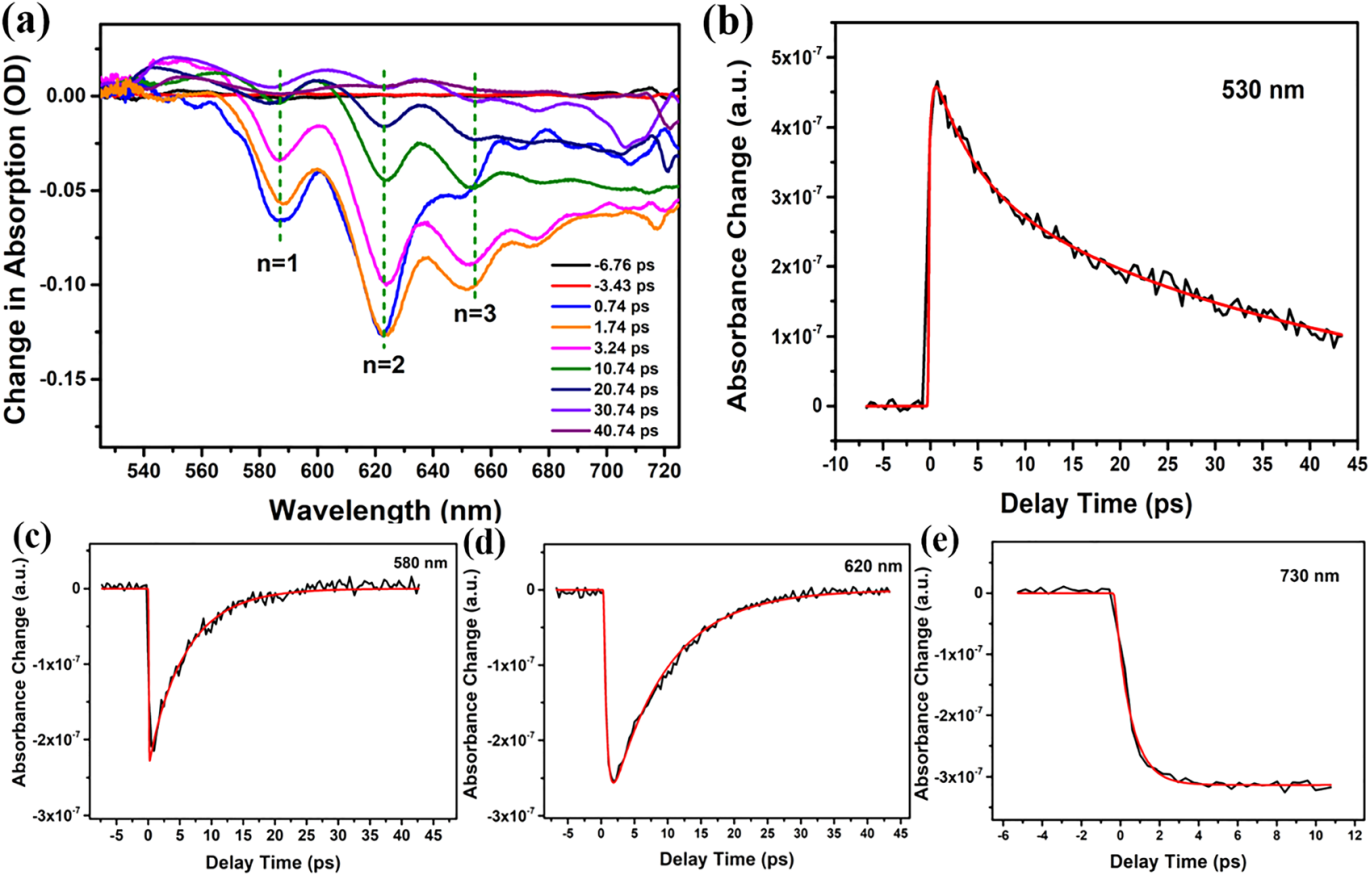
(a) TA spectra obtained for thin film ACI 3 in a 50 ps time window, (b–e) TA decay traces and corresponding fits obtained for thin film ACI 3 at 530 nm (b), 580 nm (c), 620 nm (d) and 730 nm (e) in a 50 ps time window.

Next, to gain further insight into the charge carrier dynamics of the perovskites with different numbers of layers, we performed femtosecond TAS. In these experiments, a fs pulse (pump) was used to excite the perovskite films. The changes in absorption (ΔA) were probed by a second fs pulse (probe), and the spectra were recorded at a certain delay between the two pulses. Figure 4(a) and Figure S11 show the fs TA spectra obtained for ACI 1, 2, and 3 thin films. The corresponding bleaching kinetics of ACI 3 at 530, 580, 620 and 730 nm in a 50 ps time window are illustrated in Figure 4(b–e). In these experiments, a lock-in technique (heterodyne) was used, and the obtained spectra were fitted with a multiexponential function [50], [51], [52]. All fs TA spectra are characterized by a positive and long-lived band in the wavelength range of 500–580 nm, which is formed with a time constant of 300 fs and indicates photoinduced absorption due to refractive index changes and exciton generation [53]. The band decays with time constants of ∼4.1 ps and greater than 50 ps attributed to fast exciton relaxation and dissociation (Figure 4(b)), respectively. The TA spectra also feature strong negative bands related to different QWs at 580–590 (*n* = 1), 600–620 (*n* = 2) and 640–660 nm (*n* = 3), assigned to the maximum valence band (VB) depopulation [22, 23]. The spectral positions of the photoinduced bleaching (PIB) bands correspond to the excitonic peaks observed in stationary absorption and provide information about the carrier dynamics and density. These noteworthy signals indicate that for *n* = 1, the intense PIB occurs rapidly (time constant of 130 fs at 580 nm), while for *n* = 2, this occurs slightly slower (time constant of 500 fs at 620 nm). This phenomenon is due to the dielectric confinement being reduced for the *n* > 1 systems; hence, the exciton formation time is slower (Figure 4(c and d)). For *n* > 3, the exciton formation slows down further (time constant of 810 fs), as this band at 740 nm reaches a maximum in 3 ps (Figure 4(e)). These bands indicate depopulation with time constants of 4–30 ps and can be tentatively attributed to exciton relaxation in the conduction band (Figure 4(a, c, and d)). This is also supported by the bathochromic shift of the bands (Figures 4(a) and S6, e.g., the *n* = 1 band shifts from 580 to ≈590 nm).

Earlier reports on the vertically stacked microcrystals of low-dimensional ACI perovskites assembled within 3D perovskite nanoscale networks attributed such prompt time constants to charge carrier localization from low-dimensional ACI perovskites to 3D perovskites (with a smaller bandgap) [23]. According to our data, these band depopulation decay time constants are on the order of ps or longer. For instance, when comparing the TA dynamics of ACI 3 with the ACI 1 and ACI 2, the band attributed to *n* = 1 in sample ACI 1 decay much slower than in sample ACI 3 due to a reduced presence of QW with other *n*-values (Figure 4(a), Figure S11). Interestingly, the TA spectra of ACI 3 feature additional bands located at 670 nm and higher wavelengths, indicating exciton resonances of ACI perovskites with *n* > 3. The formation of these species is even slower than that for *n* = 3 but faster than their decay. Thus, energy transfer possibly occurs towards the structural phases with a higher number of layers (possibly bulk 3D). In contrast to ACI 1 and ACI 2, the TA spectra of ACI 3 show an intense photobleaching (PB) signal in the 700–730 nm region, whose origin can be assigned to transitions between the VB and conduction band [53]. These results clearly indicate that a substantial portion of the photogenerated excitons from lower *n* QWs (high bandgap) will be gradually localized to higher *n* QWs (low bandgap) in the mixed ACI samples. In addition, this observed charge carrier localization in ACI perovskites is slightly faster than that in previously reported 2D RP perovskites [50], [51], [52]. The faster charge carrier localization may be caused by the shortened distance between the inorganic layers and the optimized special arrangement of the cations in the ACI perovskite.

### 2.3 Performance of PeLEDs fabricated with ACI perovskites

**Figure 5:**
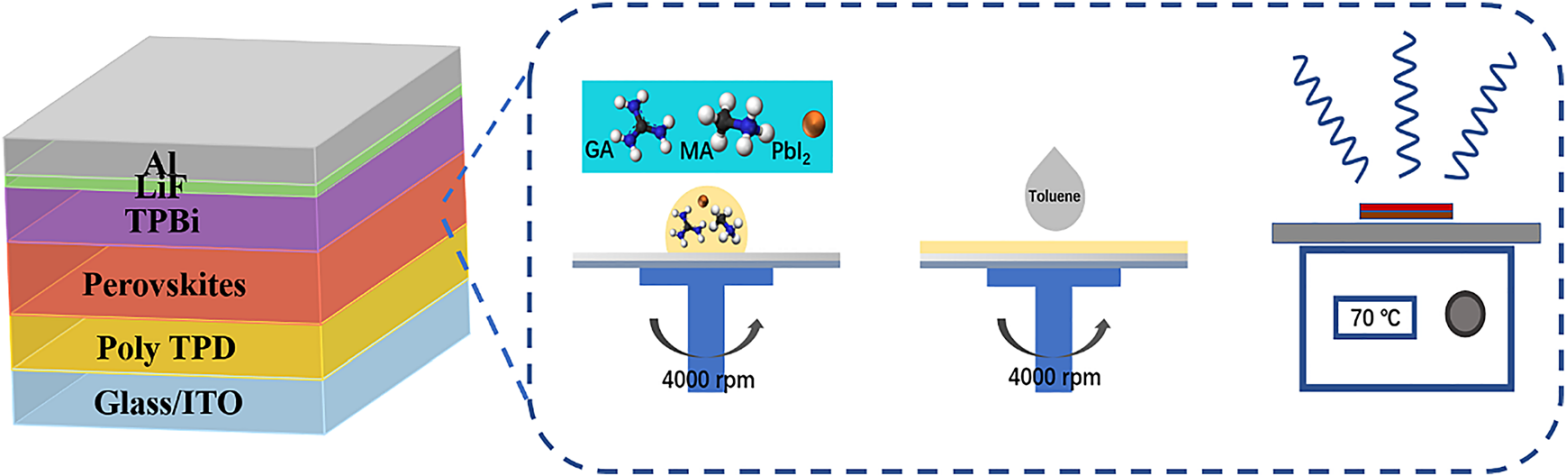
Schematic of the perovskite LED structure used in this work, along with the perovskite structure and fabrication process. The perovskite films were deposited by spin coating at 4000 rpm. An antisolvent step was performed by dropping toluene on the substrates 5 s after commencing spinning. The samples were then annealed at 70 °C for 10 min.

**Figure 6:**
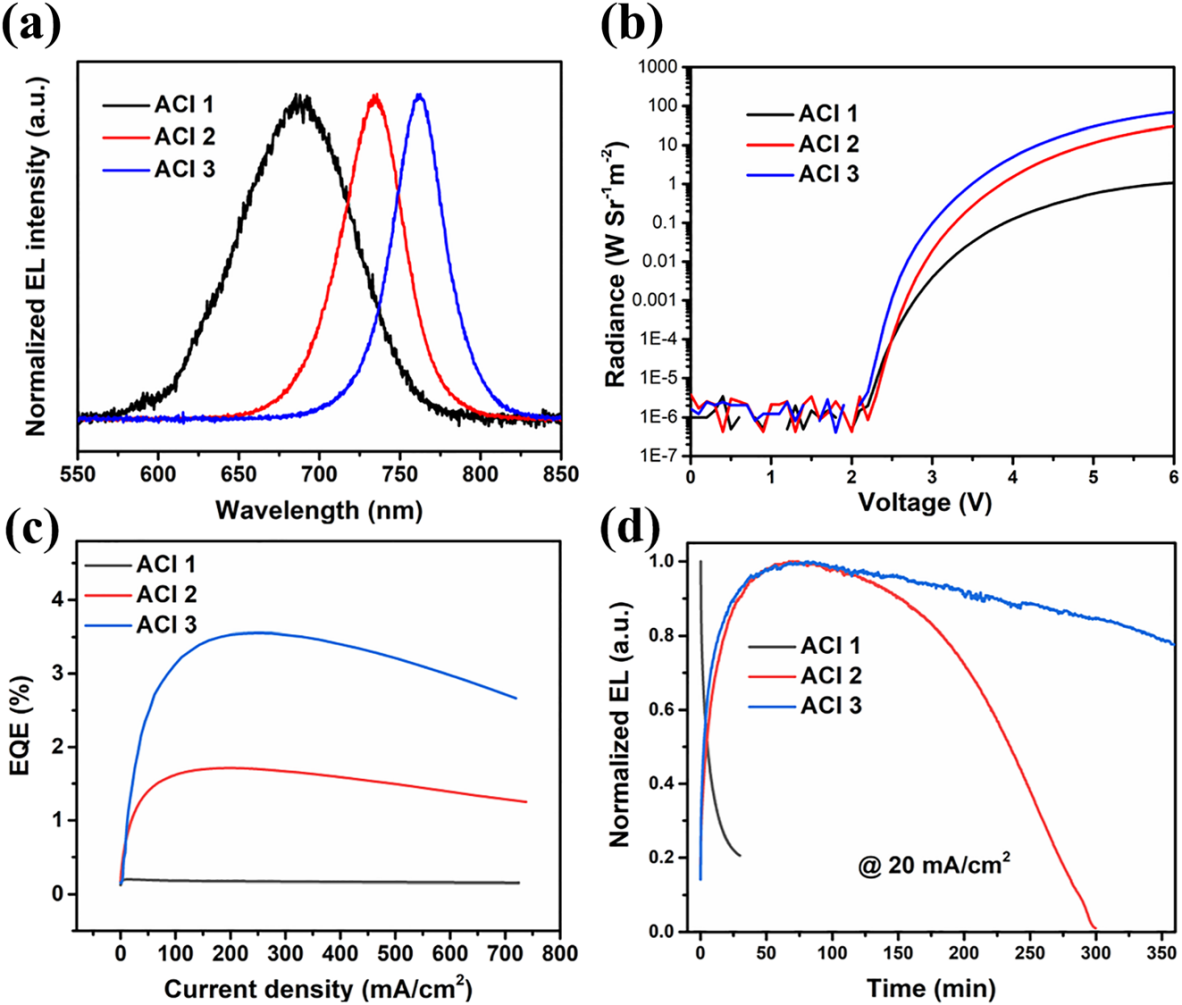
(a) EL spectra of the ACI perovskites from the corresponding devices, (b) radiance versus voltage curves of LEDs based on ACI films, (c) EQE versus current density characteristics of PeLEDs based on ACI films, (d) Normalized EL intensity of device emitting layers under a constant current density of 20 mA cm^−2^.

Finally, we evaluated the performance of the different ACI films in PeLEDs. The devices were prepared with a conventional architecture of ITO/PolyTPD/perovskite/TPBi/LiF/Al (Figure 5, together with the fabrication process [54, 55]). Figure 6(a) presents the typical electroluminescence (EL) spectra recorded from the devices made with ACI 1, ACI 2, and ACI 3, which resemble the PL spectra shown in Figure 2(b). Figure 6(b) presents the radiance-voltage curves of the corresponding devices, showing similar turn-on voltages of ∼2.1 V. The turn-on voltages are very similar for the devices employing the same device structure with PolyTPD and TPBi as the hole and electron injection layers. The radiance value increases in the sequence of ACI 1, ACI 2, and ACI 3. It reaches 77.49 W Sr^−1^ m^−2^ at 6 V for ACI 3, outperforming those of ACI 1 (1.15 W Sr^−1^ m^−2^) and ACI 2 (31.82 W Sr^−1^ m^−2^) at the same voltage. The radiance increases sharply once the voltage reaches the threshold voltage, demonstrating a low series resistance and efficient carrier injection into the device [19]. According to the EQE-current density (EQE-J) curves, the ACI 3 device achieves a maximum EQE of 3.4% at 249 mA cm^−2^, while the maximum EQEs of the ACI 1 and ACI 2 devices are 1.56 and 0.11%, respectively. The relatively high EQE can be maintained in a wide range of current densities, e.g., it remains above 2.5% for J up to 720 mA cm^−2^ (Figure 6(c)). The low efficiency roll-off is superior to most of the reported perovskite LED devices based on hybrid perovskite structure [56]. The better device performance of ACI 3 agrees with its shorter carrier lifetime, more uniform film, higher PL quantum yield (PLQY), more efficient carrier funneling, and higher exciton binding energy than typical 3D perovskites. More encouragingly, the ACI 3 device maintains over 80% of its initial performance after operating at 20 mA/cm^2^ for 350 min (Figure 6(d)), outperforming devices based on other types of perovskites with the same device structure [7, 8, 17, 33, 57]. In Table S3, we summarize the EL peak wavelength, maximum EQE, operation stability and test conditions at a constant current density of the thin film LEDs in this work as well as other thin films in previous literature. We note that the emission spectrum also remains the same before and after bias application, indicating the excellent phase stability (Figure S12). Therefore, we believe that ACI perovskites with further optimized compositions are promising candidates to achieve highly stable and efficient PeLEDs.

## 3 Conclusion

In summary, 2D ACI perovskites have been applied in PeLEDs as the active layer for the first time. The morphological and optoelectronic properties of the ACI perovskites were investigated for varying *n* between 1 and 3. Our results indicate that the ACI 3 perovskite film has favorable characteristics for PeLEDs. This is confirmed by the PeLED device performance. We achieve the highest EQE of 3.4% for devices using ACI 3 perovskites. Such devices also show encouraging operational stability, maintaining 80% of the initial performance and without changes in the emission spectrum after being biased at 20 mA/cm^2^ for 350 min. Our results demonstrate that 2D ACI perovskites are an alternative platform for further investigation to achieve high-efficiency and stable PeLEDs.

## 4 Experimental section

### 4.1 Device structure

The near-infrared (NIR) emission LEDs were made with a device structure of ITO/poly[N,N′-bis(4-butylphenyl)-N,N-bis(phenyl)-benzidine] (PolyTPD)/perovskite/1,3,5-tris(1-phenyl-1H-benzimidazol-2-yl)benzene (TPBi)/LiF/Al (Figure 2(a)).

### 4.2 Materials

Lead iodide (PbI_2_, 99%) powder, methylammonium iodide (MAI, 99%) powder, guanidine iodide (GAI, 99%) powder, toluene (anhydrous, 99.8%), N,N-dimethylformamide (DMF, anhydrous, 99.8%) and dimethyl sulfoxide (DMSO, anhydrous, 99.8%) were purchased from Alfa Aesar. LiF powder was obtained from Sigma-Aldrich. PolyTPD and TPBi were purchased from American Dye Source and Lumtec, respectively. All of the commercial materials were used as received.

### 4.3 Material synthesis and film preparation

Hybrid ACI 2D perovskite (GA)(MA)_n_Pb_3n+1_ films were fabricated using one-step spin coating with antisolvent (chlorobenzene) dripping from the precursor solution. For ACI perovskites with pure iodide, GAI and MAI in different molar ratios (1–0.3:1) were dissolved together with PbI_2_ (461 mg, 1 mmol) in a mixture solution of DMF and DMSO (volume ratio, 9:1) under magnetic stirring for 24 h to obtain a 0.4 M GAMA_n_Pb_n_I_3n+1_ (*n* = 1, 2, 3) precursor solution, which was used to deposit NIR emissive ACI perovskite films. The perovskite films were deposited by spin coating at 4000 rpm. An antisolvent step was performed by dropping toluene on the substrates 5 s after commencing spinning. The samples were then annealed at 70 °C for 10 min.

### 4.4 Femtosecond TA experiments

An amplified femtosecond double optical parametric amplifier (OPA) laser system was used to generate the excitation and probe pulses. The assembly of a Ti-sapphire oscillator (Mai Tai SP, Spectra-Physics) coupled to a laser amplifier comprising a stretcher, a Ti-sapphire regenerative cavity amplifier and a compressor (Spitfire Pro 35F-XP, Spectra-Physics) was the source of 35 fs pulses (FWHM). The pulses had a bandwidth of 31 nm (FWHM), a 4 mJ pulse energy around 800 nm and a repetition rate of 1 kHz. The regenerative amplifier was energized by a *Q*-switched, diode-pumped Nd: YLF pulsed laser (Empower-30, Spectra-Physics) capable of delivering a 527 nm output beam of 30 W. The amplifier output was used to pump two identical two-stage OPAs of white-light continuum (Topas-C, Light Conversion). Each OPA was pumped with a 1 mJ pulse energy producing 60 fs (FWHM), 31 nm bandwidth (FWHM) pulses. The energy of the independently tunable excitation pulses was in the range of 100 μJ @ 500 nm, and the output could be tuned over a spectral domain from 300 to 2600 nm. A small percentage of the regenerative amplifier output was used to generate a white-light continuum that then served as probe light. This was done by focusing a small part of the 800 nm beam into a 3 mm sapphire plate to obtain white light in the 450–800 nm region. Monochromatic detection was performed using a photomultiplier tube (PMT) (Hamamatsu R928), whereas the spectra were recorded using a charge-coupled device (CCD) camera (Princeton Instrument Pixis 100) mounted at the exit ports of a 300 mm focal length spectrograph (Acton Research 2300). The entire system provides pulses with a duration of 100 fs (FWHM cross correlation between pump and probe) at a repetition rate of 1 kHz. After each experiment, the integrity of the samples was checked by recording the steady-state absorption and emission spectra.

### 4.5 Device fabrication

PolyTPD was spincoated on precleaned ITO (Colorado Concept Coatings) at 1000 rpm for 60 s, followed by thermal annealing at 150 °C for 20 min. To improve the surface wettability, the PolyTPD layer was treated with O_2_ plasma for 6 s at a power of 100 W. After that, the perovskite films were deposited in a N_2_-filled glove box as described in the perovskite film preparation section. Then, TPBi, LiF, and Al layers were thermally evaporated on top of the perovskite films sequentially, with thicknesses of 60, 1.2, and 100 nm, respectively. The device area was 0.125 cm^2^ with dimensions of 2.5 × 5 mm.

### 4.6 Characterizations

GIWAXS measurements have been performed at the Austrian SAXS beamline at the ELETTRA synchrotron in Trieste (Italy) at a photon energy of 8 keV [58]. The beam size was set to 1 × 0.1 mm^2^ (*H* × *V*) while the sample to detector (Pilatus3 1M, Dectris) distance was adjusted to 217.3 mm using a silver behenate as a reference pattern. All measurements have been performed in air at the incident angle of 0.5°. The patterns have been corrected for the fluctuations of the primary intensity [59]. The simulations of the GIWAXS patterns have been performed with the GIXSGUI toolbox [39] that has also been used for extraction of the azimuthal intensity profiles in equal Δq interval both in the proximity of (002), (004), and (006) reflections for ACI 1–3 films at lower q to be used for background estimation, respectively. The successive background subtraction from the azimuthal peak intensity profiles and calculation of the Hermans’ orientation function <*P*(*θ*)> [60] has been performed using IGOR Pro (IGOR Pro 7.0.8.1, WaveMetrics). The powder diffraction patterns have been simulated with Diamond 3.2i (Crystal Impact). AFM measurements were performed in air using a Multimode AFM with a Nanoscope VIII controller (Bruker) in tapping mode. Olympus silicon cantilevers (AC160TS-R3) with a nominal frequency resonance of 300 kHz and spring constant around 26 N m^−1^ were employed. The value for the RMS roughness for each sample consists of an average obtained from 16 different areas of 500 × 500 nm. Gwyddion software was used for AFM image processing (Nečas, D.; Klapetek, P. Gwyddion: An Open-Source Software for SPM Data Analysis. Central European Journal of Physics. January 1, 2012, pp 181–188). UV–vis diffuse reflectance spectra (UV–vis DRS) were measured using a Lambda 950 UV–vis spectrophotometer. PL measurements were performed on an Edinburgh FLS980 using a He-Cd laser with a 470 nm excitation wavelength at room temperature. Lifetime spectra were measured using Leica TCS SP8 X ‘FLIM’ measurements with a 470 nm laser at a 10 MHz repetition rate. Temperature-dependent PL experiments were performed using a Thorlabs M365LP1 solid-state laser with a DC2200 driver operating at 365 nm. The sample was excited via a 550 μm core optical fiber, and the PL spectra were collected through 11 core optical fibers of 200 μm diameter that surrounded the excitation fiber. The detection fibers were coupled to a LOT-QD Shamrock F/4 spectrometer with an iXon DV887 electron multiplying CCD (EMCCD). The spectra were collected every 5 K between 4.2 and 300 K in a He flow cryostat. A Thorlabs integrating sphere (IS236A-4) coupled with a calibrated silicon photodiode (SM05PD1B) and a Flame Spectrometer from Ocean Optics was used to measure the EL intensity and the emission spectrum. The response of the photodiode was calibrated together with the integrating sphere by Thorlabs. The J–V-R characteristics were measured by an Agilent 4156C Semiconductor Parameter Analyzer which also records the corresponding photodiode current at the same time.

## Supplementary Material

Supplementary Material

Supplementary Material
