# Supplementary material for: Two-dimensional perovskites with alternating cations in the interlayer space for stable light-emitting diodes
Source: Nanophotonics. 2021 Apr 29;10(8):2145–56. doi: 10.1515/nanoph-2021-0037 (PMC9646242; doi:10.1515/nanoph-2021-0037)
Supplement: Supplementary file 1 — Supplementary Material [file j_nanoph-2021-0037_suppl_001.docx]

Yiyue Zhang, Masoumeh Keshavarz, Elke Debroye, Eduard Fron, Miriam Candelaria Rodríguez González, Denys Naumenko, Heinz Amenitsch, Joris Van de Vondel, Steven De Feyter, Paul Heremans, Maarten B.J. Roeffaers, Weiming Qiu*, Bapi Pradhan*, Johan Hofkens*

Two-dimensional Perovskites with Alternating Cations in the Interlayer Space for Stable Light-Emitting Diodes

***Corresponding authors:**

***Johan Hofkens****, *Bapi Pradhan, *Weiming Qiu:**

Molecular Imaging and Photonics, Department of Chemistry, KU Leuven, Celestijnenlaan 200F, Leuven 3001, Belgium.

***Johan Hofkens:**

Max-Planck-Institute for Polymer Research, Mainz 55128, Germany

**E-mail:** [johan.hofkens@kuleuven.be](mailto:johan.hofkens@kuleuven.be); [bapi.pradhan@kuleuven.be](mailto:bapi.pradhan@kuleuven.be) [weiming.qiu@imec.be](mailto:weiming.qiu@imec.be)

**Yiyue Zhang, Eduard Fron, Masoumeh Keshavarz, Elke Debroye, Miriam Candelaria Rodríguez González and Steven De Feyter:**

Molecular Imaging and Photonics, Department of Chemistry, KU Leuven, Celestijnenlaan 200F, Leuven 3001, Belgium.

**Joris Van de Vondel:**

Quantum Solid-State Physics (QSP), Department of Physics and Astronomy, KU Leuven, Celestijnenlaan 200D, Leuven 3001, Belgium.

**Maarten B.J. Roeffaers:**

cMACS, Department of Microbial and Molecular Systems, KU Leuven, Celestijnenlaan 200F, Leuven 3001, Belgium

**Paul Heremans, *Weiming Qiu:**

Imec, Kapeldreef 75, 3001 Leuven, Leuven

**Denys Naumenko, Heinz Amenitsch:**

Institute of Inorganic Chemistry, Graz University of Technology, Stremayrgasse 9/V, Graz, 8010, Austria

**Table of Contents**

1. GIXRD of ACI perovskites…………………...……………………………...…….Figure S1

2. Crystal data and refined lattice parameters of ACI perovskites…………………….Table S1

3. Crystal orientation from GIWAXS...………………………...………...….…….…Figure S2

4. High resolution atomic force microscopy……………………………………....….Figure S3

5. UV-vis spectra of ACI perovskites……………………………………...…….……Figure S4

6. Optical images of the ACI films……………………….……………….….……....Figure S5

7. Bandgap calculation of ACI perovskites……………………………….…….…....Figure S6

8. Excitation-emission map of ACI perovskites……………………………….……...Figure S7

9. PL spectra of ACI samples with phase separation…………………….…….…….Figure S8

10. Fluorescence decay time constants………….………………………….………….Table S2

11. Transient absorption spectra for ACI 1 and ACI 2……………………….……....Figure S9

12. LED devices parameters comparison………...…………………….…….….…….Table S3

13. Normalized EL spectrum before and after bias…………………………….…....Figure S10


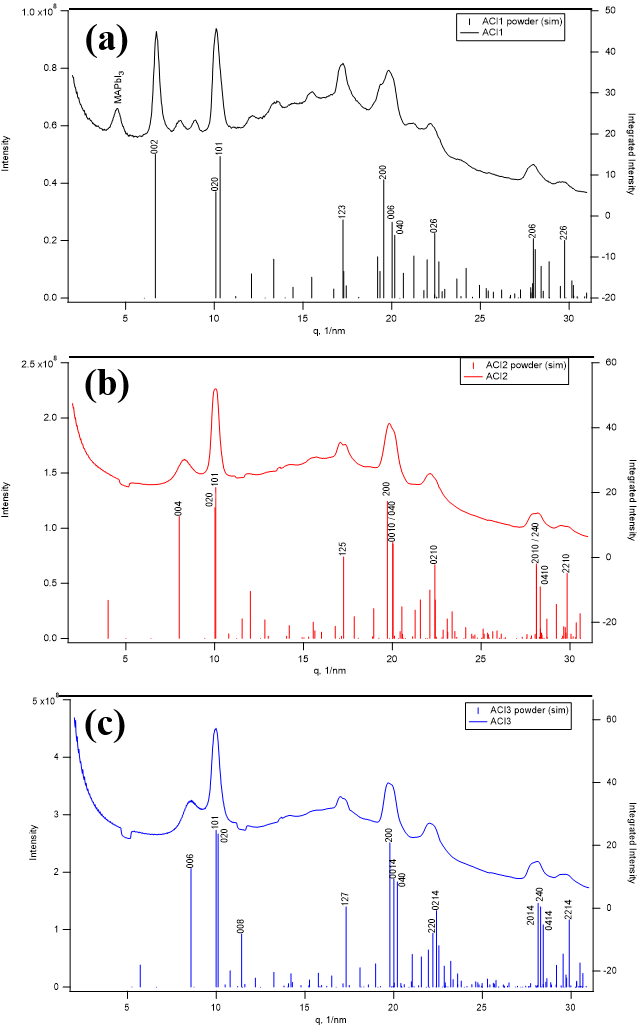


**Figure S1.** (GA)(MA)_n_Pb_n_I_3n+1_ (ACI n, n = 1-3) simulated powder diffraction using single crystal data from[1]. Right vertical axes represent an azimuthal integration of GIXRD data on ACI films shown in the main text in Figure 1a-c. All identified peaks in the GIXRD patterns are highlighted in the powder diffraction data and exceed 30% of maximum simulated intensity value. The peak at q = 4.5 nm^-1^ for ACI 1 film is attributed to MAPbI_3_ phase.

**Table S1.** Crystal data of (GA)(MA)_n_Pb_n_I_3n+1_ (ACI n, n = 1-3) used for powder diffraction simulations[1] and refined lattice parameters obtained from GIXRD analysis on corresponding films with c axis oriented perpendicularly to the substrate. The Asterisk highlights changes of lattice parameter b with b axis perpendicular and c axis parallel to the substrate.

| sample | a, A | b, A | c, A | Space group |
| --- | --- | --- | --- | --- |
| ACI 1 (sim) | 6.43 | 12.46 | 18.83 | (74) Imma (O) |
| ACI 1 | 6.43 | 12.47 (12.85*) | 19.80 | (74) Imma (O) |
| ACI 2 (sim) | 6.37 | 12.54 | 31.38 | (38) Bmm2 (O) |
| ACI 2 | 6.40 | 12.55 (12.90*) | 33.00 | (38) Bmm2 (O) |
| ACI 3 (sim) | 6.35 | 12.44 | 43.97 | (74) Imma (O) |
| ACI 3 | 6.38 | 12.60 (12.8*) | 46.30 | (74) Imma (O) |


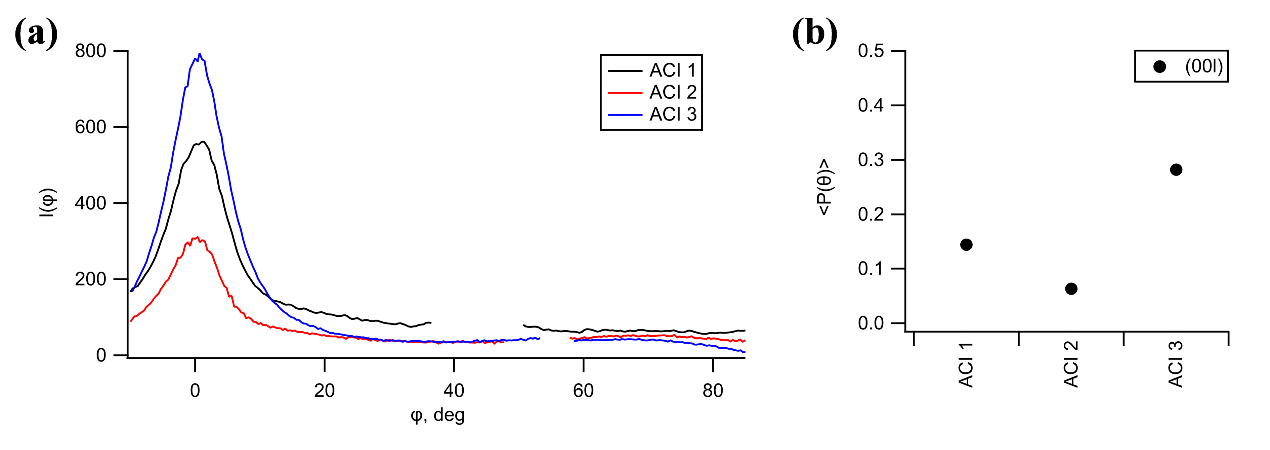


**Figure S2.** (a) The background corrected scattered intensity I(φ) as a function of the azimuthal angle φ integrated in the proximity of (002), (004), and (006) reflections for ACI n, n =1-3 films, respectively. (b) The calculated Hermans’ orientation function <P(θ)> for ACI 1-3 films.


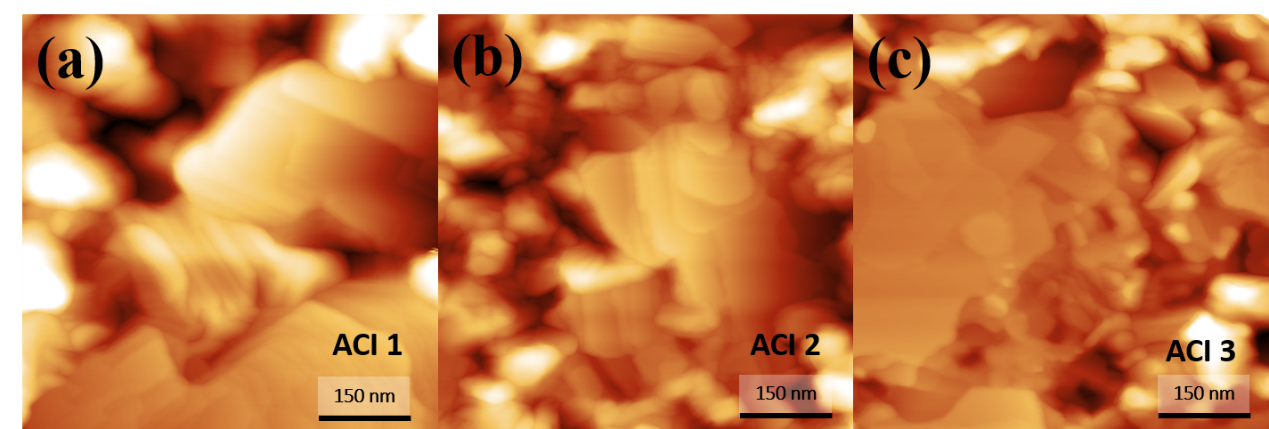


**Figure S3.** High resolution atomic force microscopy (AFM) images (600 nm x 600 nm) of ACI 1, 2 and 3 films.





**Figure S4.** The UV–vis absorption spectra of ACI perovskites at 550-700 nm. fundamental exciton n = 1 at 1.9 eV (654 nm), n = 2 at 2 eV (623 nm), and n = 2.14 eV (580 nm), representing different quantum well (QWs) structures.


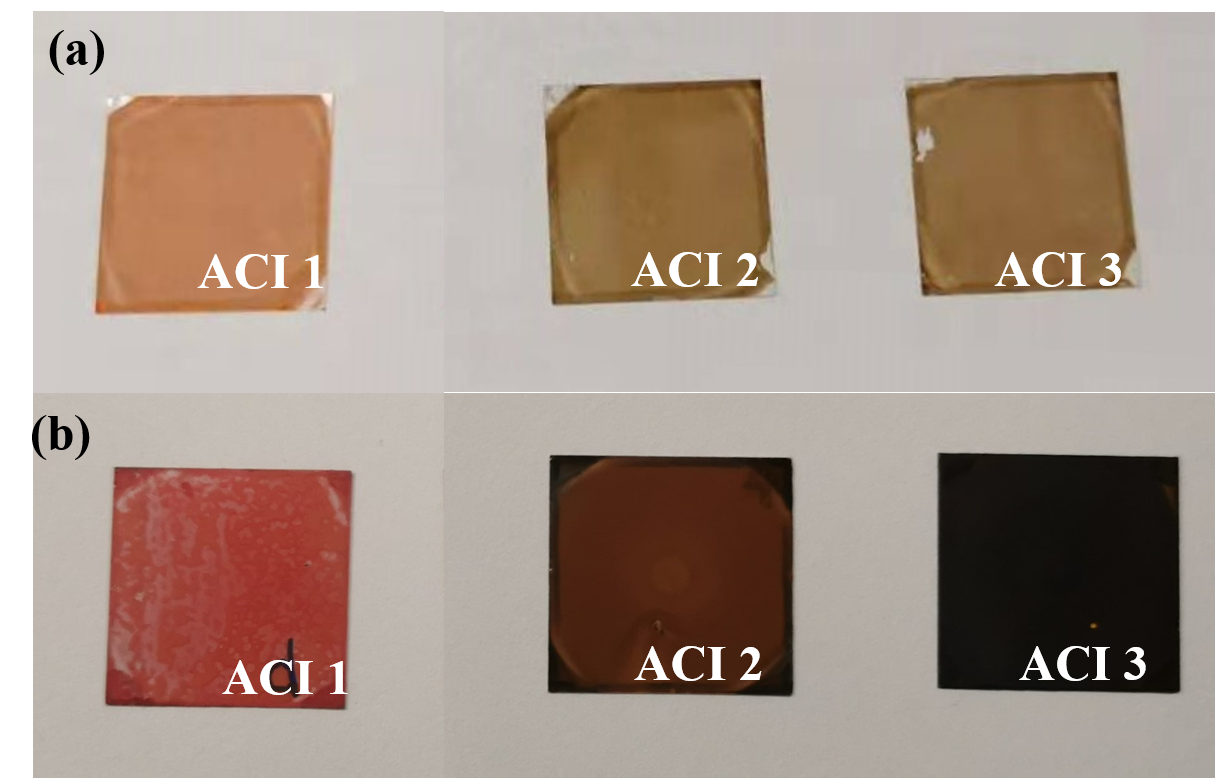


**Figure S5.** The optical images of the (GA)(MA)_n_Pb_n_I_3n+1_ films. a) thin film (0.2M precursor) b) thick film (1M precursor).





**Figure S6.** Direct bandgap calculation of ACI perovskites extracted from Kubelka-Munk analysis. According to the Kubelka-Munk formula: K/S=(1-R)^2^/2R= B(hν-E_g_). Where the K is absorption coefficient, S is the reflection coefficient, R is reflectivity (%), h is Planck's constant, ν is the frequency of the light, E_g_ is a physical quantity related to the material, that is, the energy bandgap, n=1/2 is for indirect bandgap semiconductor, n=2 is for direct bandgap semiconductor. By plotting K/S vs hν, the obtained intersection on the x axis is the bandgap energy.


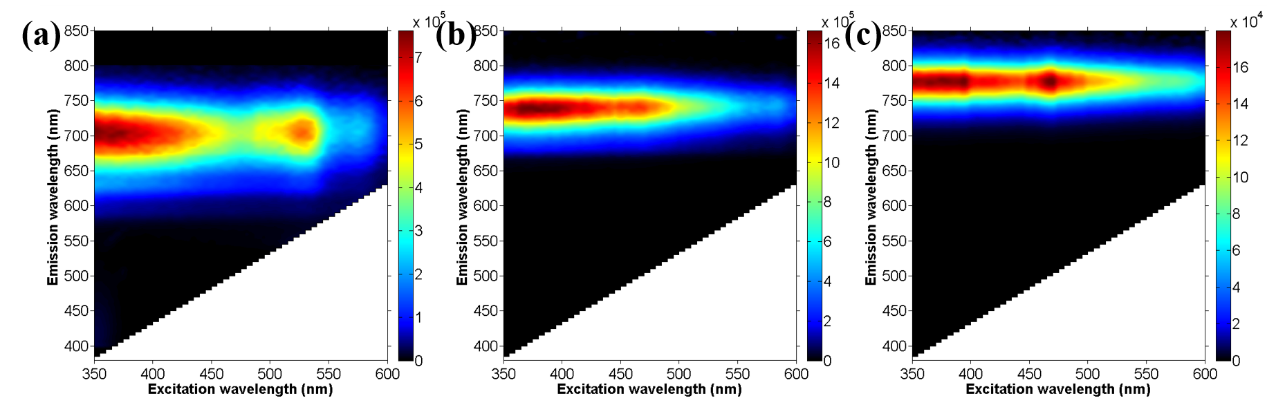


**Figure S7.** The excitation-emission map of a) ACI 1, b) ACI 2, and c) ACI 3 films.





**Figure S8.** The PL spectra of ACI samples with phase separation caused by changes in related experimental parameters. (without anti-solvent, without DMSO, with excess GA content).


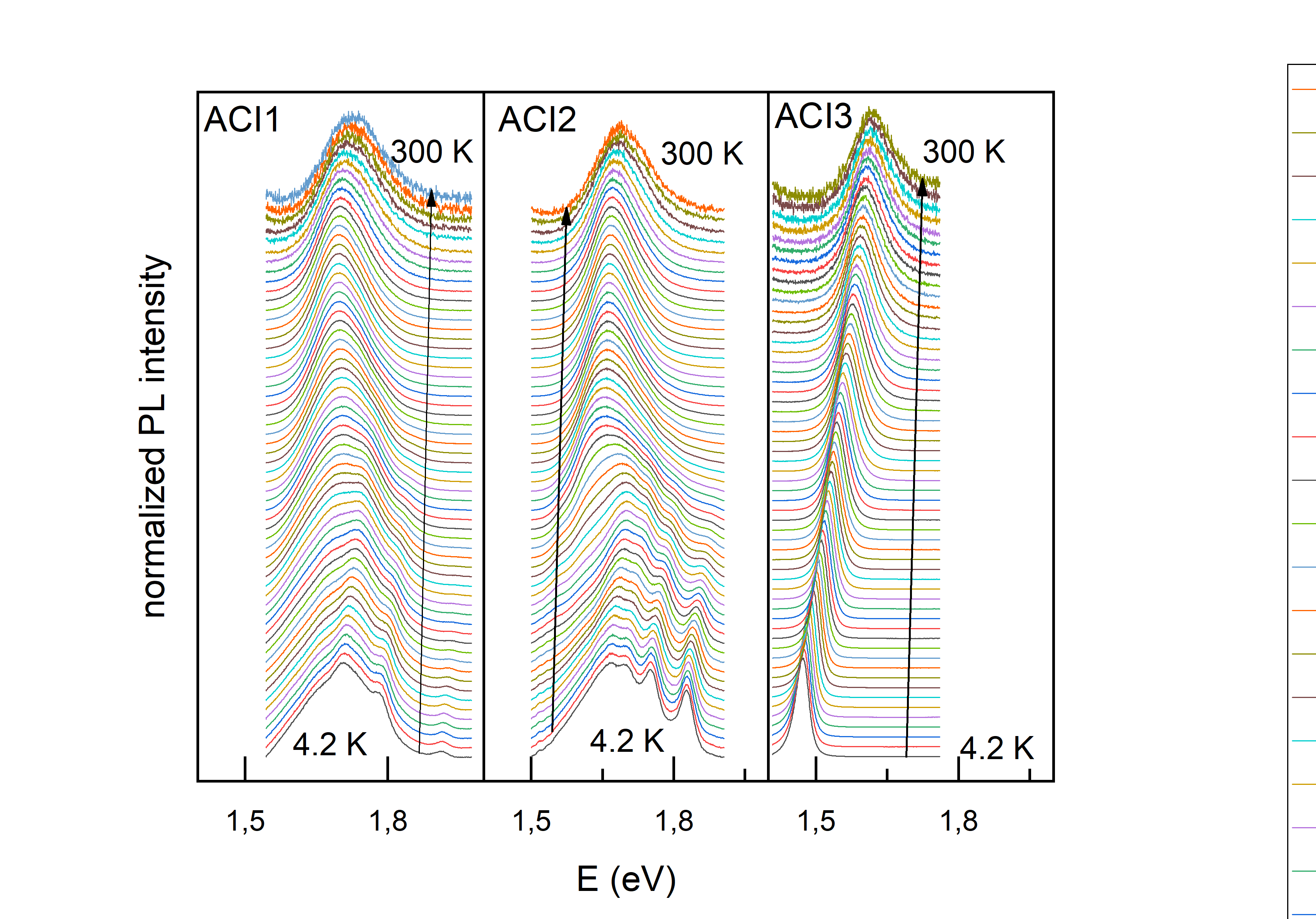


**Figure S9**. Temperature evolution of the PL spectra for all three ACI materials between 4.2 and 300 K collected at an increment of 5K.


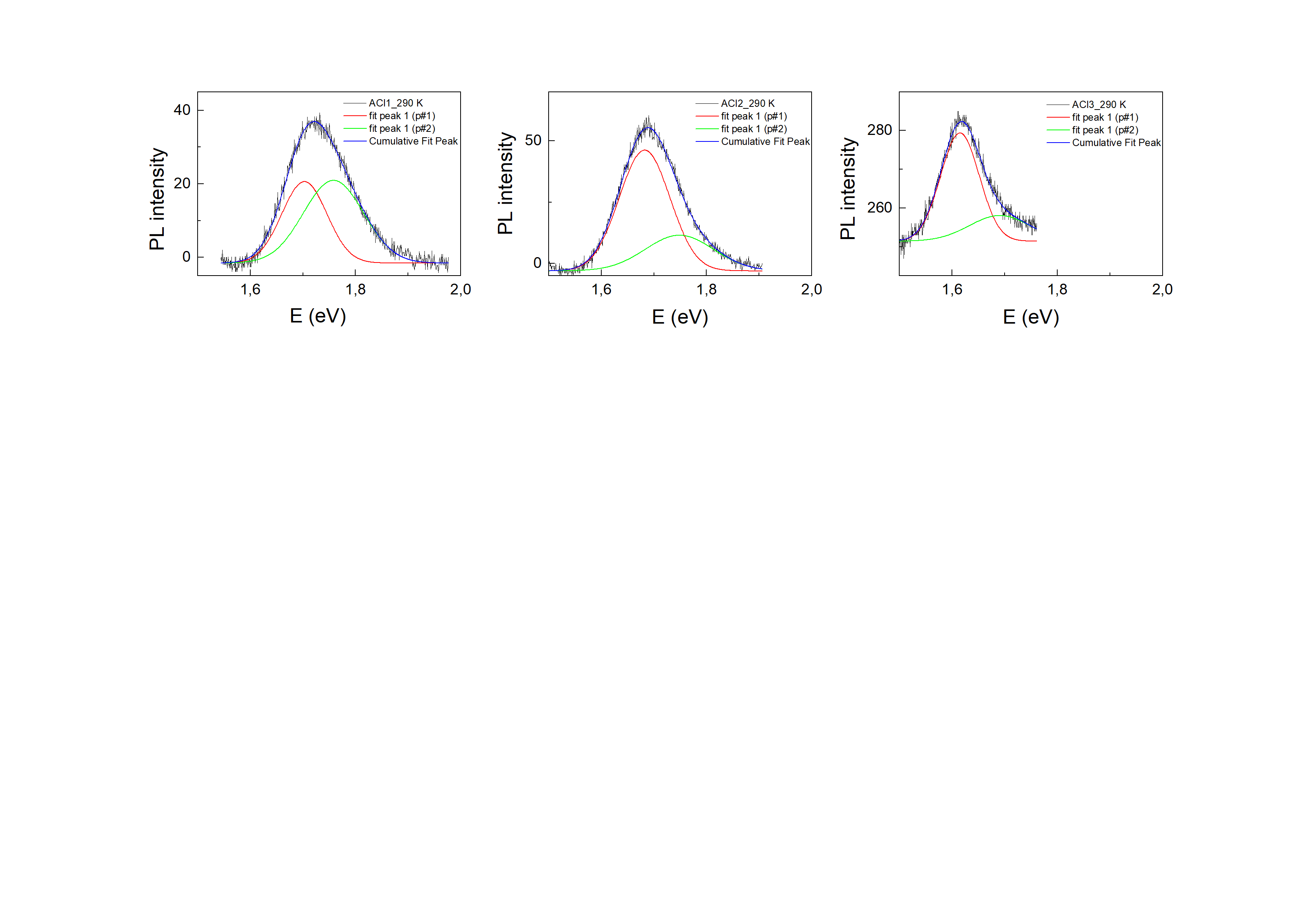


**Figure S10**. The PL emission spectra of all three ACI materials at room temperature fitted with a multipeak Gaussian function (two peaks here). The fitted peaks are abbreviated as p#1 and p#2 for each material and are used for further analysis throughout the manuscript.

**Table S2.** Fluorescence decay time constants obtained from multiexponential decay trace fitting of the TCSPC data. Excitation wavelengths have been set to 470 nm.

|  | **ACI 1** | **ACI 2** | **ACI 3** |
| --- | --- | --- | --- |
| τ_[1]_ [ns] | 44.41 | 36.8 | 22.48 |
| τ_[2]_ [ns] | 4.35 | 5.2 | 3.56 |
| τ_[3]_ [ns] | 0.29 | 0.5 | 0.43 |


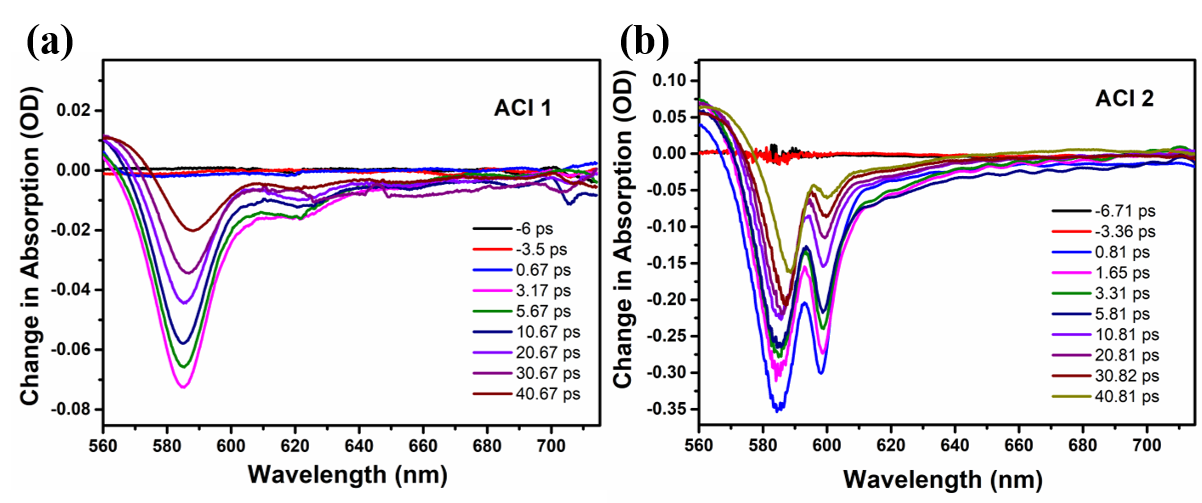


**Figure S11.** Transient absorption spectra obtained for thin film ACI 1 (a) and ACI 2 (b) in 50 ps time window.

**Table S3.** Summary of EL peak wavelength, maximum EQE, and operation stability at constant current density of thin film LEDs in this work and in the literature.

| Materials Emitter | λ_EL_ | EQE_max_ [%] | | Stability | Ref | |
| --- | --- | --- | --- | --- | --- | --- |
| GAMA_3_PbI_10_ (ACI 3) | **770** | **3.4** | **6 h @ 20 mA cm^–2^ (EQE_80_)** | | **This work** |  |
| 5AVAFA_n-1_Pb_3_I_3n+1_ | 800 | 20.1 | 200 min @ 100 mA cm^–2^ (EQE_80_) | | [2] |  |
| FAPbI_3_ | 770 | 5.1 | 80 min @ 10 mA cm^–2^ (EQE_80_) | | [3] |  |
| Cs_0.2_FA_0.8_PbI_2.8_Br_0.2_ | 760 | 11.6 | 70 min @ 10 mA cm^–2^ (EQE_80_) | | [3] |  |
| MAPbI_3_ (thin film) | 757 | 10.6 | 3.5h @ 10 mA cm^–2^ (EQE_50_) | | [3] |  |
| MAPbI_3_ (thick film) | 752 | 4.8 | 3h @ 10 mA cm^–2^ (EQE_50_) | | [3] |  |
| BAB-FAPbI_3_ | 750 | 4.3 | 40h @ 25 mA cm^−2^ (EQE_50_) | | [4] |  |
| BAMA_5_PbI_8_ | 750 | 3.6 | ＞10 min @ 5 V (EQE_50_) | | [5] |  |
| MAPbI_3_/BAI | 748 | 10.4 | 5 h @ 3 mA cm^−2^ (EQE_75_) | | [5] |  |
| BA_2_MA_4_Pb_5_I_16_ | 744 | 0.5 | 14 h @ 2 V (>T90) | | [6] |  |
| BAMA_2_PbI_5_ | 708 | 2.1 | ＞90 s @ 5 V (EQE_50_) | | [5] |  |
| CsPb_2_I_5_ | 690 | 0.14 | 2h @10 mA cm^–2^ (T_50_) | | [7] |  |
| CsPbI_3_ | 645 | 14.1 | 180 min @ 1.25 mA cm^−2^ (T_50_) | | [8] |  |
| FA_0.9_GA_0.1_PbBr_3_ PNCs | 545 | 23.2 | 100min @ 3V (T_50_) | | [9] |  |
| FAPbBr_3_ | 535 | 11.3 | 5 min @ 10 mA cm^–2^ (EQE_50_) | | [3] |  |
| PEA_2_(FAPbBr_3_)_2_PbBr_4_ | 532 | 14.3 | 60 min @ 0.5 mA cm^−2^ (T_50_) | | [10] |  |
| PEA_2_FA_2_Pb_3_I_10_ | 530 | 10 | 2h @10 mA cm^–2^ (T_50_) | | [11] |  |
| CsPbBr_3_/MABr | 525 | 20.3 | 10.42 min @ L_0_ = 7130 cd m^−2^ (T_50_) | | [12] |  |
| MAPbBr_3_ | 524 | 12.3 | 6 min @ 0.3 mA cm^−2^ (T_50_) | | [13] |  |
| CsPb_2_Br_5_ | 520 | 1.1 | 6h @ 10 mA cm^–2^ (T_50_) | | [7] |  |
| Cs_0.87_MA_0.13_PbBr_3_ | 520 | 10.3 | 16.5 min @ 5 V (T_50_) | | [14] |  |
| PEA_2_Cs_n−1_Pb_n_Br_3n+1_ | 514 | 15.5 | 60 min @ 2 mA cm^−2^ (EQE_50_) | | [15] |  |
| MAPbBr_3_/BABr | 513 | 9.3 | 40 min @ 3 mA cm^−2^ (EQE_50_) | | [5] |  |
| PEA_2_(Rb_0.6_Cs_0.4_)_2_Pb_3_Br_10_ | 490 | 1.48 | 18.7 min @ 4.5 V (T_50_) | | [16] |  |
| CsPbBr_3_/PEACl/YCl_3_ | 485 | 11 | 80 min @ 3.2V (T_50_) | | [17] |  |
| PEA_2_Cs_n−1_Pb_n_(Br/Cl)_3n+1_ | 480 | 5.7 | 10 min @ 4.4 V (EQE_50_) | | [18] |  |
| CsPbCl_x_Br_3 − x_ | 470 | 2.1 | 10 min @ 0.5 mA cm^−2^ (T_50_) | | [19] |  |

Note that: Half-lifetime (T_50_) was defined as the time required for the luminance to decease to 50% of the start luminance (L0); EQE_50/80_ is defined as the EQE value decrease to 50%/80% from the start.





**Figure S12.** The normalized electroluminescence spectrum before and after bias.

References

[1] C. M. M. Soe, C. C. Stoumpos, M. Kepenekian et al., "New Type of 2D Perovskites with Alternating Cations in the Interlayer Space, (C(NH2)3)(CH3NH3)nPbnI3n+1: Structure, Properties, and Photovoltaic Performance," *J. Am. Chem. Soc.*, vol. 139, pp. 16297-16309, 2017.

[2] Y. Cao, N. Wang, H. Tian et al., "Perovskite light-emitting diodes based on spontaneously formed submicrometre-scale structures," *Nature*, vol. 562, pp. 249-253, 2018.

[3] L. Zhao, K. M. Lee, K. Roh et al., "Improved Outcoupling Efficiency and Stability of Perovskite Light-Emitting Diodes using Thin Emitting Layers," *Adv. Mater.*, vol. 31, pp. e1805836, 2019.

[4] Y. L. Yuequn Shang, Qi Wei et al., "Highly stable hybrid perovskite light-emitting diodes based on Dion-Jacobson structure," *Sci. Adv.*, vol. 5, pp. eaaw8072, 2019.

[5] Z. Xiao, R. A. Kerner, L. Zhao et al., "Efficient perovskite light-emitting diodes featuring nanometre-sized crystallites," *Nat. Photonics*, vol. 11, pp. 108-115, 2017.

[6] H. Tsai, W. Nie, J.-C. Blancon et al., "Stable Light-Emitting Diodes Using Phase-Pure Ruddlesden-Popper Layered Perovskites," *Adv. Mater.*, vol. 30, pp. 1704217, 2018.

[7] C. Qin, T. Matsushima, A. S. D. Sandanayaka et al., "Centrifugal-Coated Quasi-Two-Dimensional Perovskite CsPb2Br5 Films for Efficient and Stable Light-Emitting Diodes," *J. Phys. Chem. Lett.*, vol. 8, pp. 5415-5421, 2017.

[8] T. Chiba, Y. Hayashi, H. Ebe et al., "Anion-exchange red perovskite quantum dots with ammonium iodine salts for highly efficient light-emitting devices," *Nat. Photonics*, vol. 12, pp. 681-687, 2018.

[9] Y.-H. Kim, S. Kim, A. Kakekhani et al., "Comprehensive defect suppression in perovskite nanocrystals for high-efficiency light-emitting diodes," *Nature Photon.*, vol. pp. 2021.

[10] X. Yang, X. Zhang, J. Deng et al., "Efficient green light-emitting diodes based on quasi-two-dimensional composition and phase engineered perovskite with surface passivation," *Nat. Commun.*, vol. 9, pp. 570, 2018.

[11] W. Bi, Q. Cui, P. Jia et al., "Efficient Quasi-Two-Dimensional Perovskite Light-Emitting Diodes with Improved Multiple Quantum Well Structure," *ACS Appl. Mater. Interfaces*, vol. 12, pp. 1721-1727, 2020.

[12] K. Lin, J. Xing, L. N. Quan et al., "Perovskite light-emitting diodes with external quantum efficiency exceeding 20 per cent," *Nature*, vol. 562, pp. 245-248, 2018.

[13] F. Yan, J. Xing, G. Xing et al., "Highly Efficient Visible Colloidal Lead-Halide Perovskite Nanocrystal Light-Emitting Diodes," *Nano Lett.*, vol. 18, pp. 3157-3164, 2018.

[14] L. Zhang, X. Yang, Q. Jiang et al., "Ultra-bright and highly efficient inorganic based perovskite light-emitting diodes," *Nat. Commun.*, vol. 8, pp. 15640, 2017.

[15] M. Ban, Y. Zou, J. P. H. Rivett et al., "Solution-processed perovskite light emitting diodes with efficiency exceeding 15% through additive-controlled nanostructure tailoring," *Nat. Commun.*, vol. 9, pp. 3892, 2018.

[16] Y. Jiang, C. Qin, M. Cui et al., "Spectra stable blue perovskite light-emitting diodes," *Nat. Commun.*, vol. 10, pp. 1868, 2019.

[17] Q. Wang, X. Wang, Z. Yang et al., "Efficient sky-blue perovskite light-emitting diodes via photoluminescence enhancement," *Nat. Commun.*, vol. 10, pp. 5633, 2019.

[18] Z. Li, Z. Chen, Y. Yang et al., "Modulation of recombination zone position for quasi-two-dimensional blue perovskite light-emitting diodes with efficiency exceeding 5," *Nat. Commun.*, vol. 10, pp. 1027, 2019.

[19] S. Hou, M. K. Gangishetty, Q. Quan et al., "Efficient Blue and White Perovskite Light-Emitting Diodes via Manganese Doping," *Joule*, vol. 2, pp. 2421-2433, 2018.
