# Supplementary material for: Two-dimensional perovskites with alternating cations in the interlayer space for stable light-emitting diodes
Source: Nanophotonics. 2021 Apr 29;10(8):2145–56. doi: 10.1515/nanoph-2021-0037 (PMC9646242; doi:10.1515/nanoph-2021-0037)
Supplement: Supplementary file 2 — Supplementary Material [file j_nanoph-2021-0037_suppl_002.docx]

Yiyue Zhang, Masoumeh Keshavarz, Elke Debroye, Eduard Fron, Miriam Candelaria Rodríguez González, Denys Naumenko, Heinz Amenitsch, Joris Van de Vondel, Steven De Feyter, Paul Heremans, Maarten B.J. Roeffaers, Weiming Qiu*, Bapi Pradhan*, Johan Hofkens*

Two-dimensional Perovskites with Alternating Cations in the Interlayer Space for Stable Light-Emitting Diodes

***Corresponding authors:**

***Johan Hofkens****, *Bapi Pradhan, *Weiming Qiu:**

Molecular Imaging and Photonics, Department of Chemistry, KU Leuven, Celestijnenlaan 200F, Leuven 3001, Belgium.

***Johan Hofkens:**

Max-Planck-Institute for Polymer Research, Mainz 55128, Germany

**E-mail:** [johan.hofkens@kuleuven.be](mailto:johan.hofkens@kuleuven.be); [bapi.pradhan@kuleuven.be](mailto:bapi.pradhan@kuleuven.be) [weiming.qiu@imec.be](mailto:weiming.qiu@imec.be)

**Yiyue Zhang, Masoumeh Keshavarz, Elke Debroye, Eduard Fron, Miriam Candelaria Rodríguez González and Steven De Feyter:**

Molecular Imaging and Photonics, Department of Chemistry, KU Leuven, Celestijnenlaan 200F, Leuven 3001, Belgium.

**Joris Van de Vondel:**

Quantum Solid-State Physics (QSP), Department of Physics and Astronomy, KU Leuven, Celestijnenlaan 200D, Leuven 3001, Belgium.

**Maarten B.J. Roeffaers:**

cMACS, Department of Microbial and Molecular Systems, KU Leuven, Celestijnenlaan 200F, Leuven 3001, Belgium

**Paul Heremans, *Weiming Qiu:**

Imec, Kapeldreef 75, 3001 Leuven, Leuven

**Denys Naumenko, Heinz Amenitsch:**

Institute of Inorganic Chemistry, Graz University of Technology, Stremayrgasse 9/V, Graz, 8010, Austria

**Abstract:** Lead halide perovskites have attracted tremendous attention in photovoltaics due to their impressive optoelectronic properties. However, the poor stability of perovskite-based devices remains a bottleneck for further commercial development. Two-dimensional perovskites have great potential in optoelectronic devices, as they are much more stable than their three-dimensional counterparts and rapidly catching up in performance. Herein, we demonstrate high-quality two-dimensional novel perovskite thin films with alternating cations in the interlayer space. This innovative perovskite provides highly stable semiconductor thin films for efficient near-infrared light-emitting diodes (LEDs). Highly efficient LEDs with tunable emission wavelengths from 680 to 770 nm along with excellent operational stability are demonstrated by varying the thickness of the interlayer spacer cation. Furthermore, the best-performing device exhibits an external quantum efficiency of 3.4% at a high current density (J) of 249 mA/cm^2^ and remains above 2.5% for a J up to 720 mA cm^−2^, leading to a high radiance of 77.5 W/Sr m^2^ when driven at 6 V. The same device also shows impressive operational stability, retaining almost 80% of its initial performance after operating at 20 mA/cm^2^ for 350 minutes. This work provides fundamental evidence that this novel alternating interlayer cation 2D perovskite can be a promising and stable photonic emitter.

**Keywords:** two-dimensional perovskites, alternating interlayer cations, light-emitting diodes, stability

**1 Introduction**

Two-dimensional (2D) metal halide perovskites have recently attracted broad attention due to their impressive optoelectronic properties for various applications[1-3], such as solar cells[4, 5], photodetectors[6] and light-emitting diodes (LEDs)[7-9]. To date, 2D layered Ruddlesden-Popper (RP) phase perovskites are the most common structural type that has been intensively studied. Generally, they have demonstrated superior stability against humidity compared to 3D perovskites[10-12] since the long organic spacer cations in RP perovskites make them more hydrophobic, preventing moisture induced degradation. Furthermore, the high dielectric contrast between the inorganic slab and the organic spacer hence, the presence of a dielectric quantum confinement results in a much larger exciton binding energy compared to 3D perovskites[7], leading to enhanced radiative recombination that is beneficial for LED applications. Indeed, perovskite LEDs (PeLEDs) based on RP perovskites have been demonstrated to have high external quantum efficiencies (EQEs) over 20% in recent years[13-17]. The operational stability of these devices, however, is unfortunately far from satisfactory, with half-lives ranging from only a few minutes to a few hours depending on the electrical bias. In addition, the long chain spacer cation has been proven to severely hamper charge transport within the device[4, 18]. Therefore, a high driving voltage is required which is unfavorable for prolonged device operational stability. Another type of 2D perovskite, i.e., the Dion-Jacobson (DJ) structure, was introduced by Ning *et al*[19] and has been used in PeLEDs. Due to the higher binding energy between the layered structures and higher molecular dissociation energy of the DJ structure than that of the RP structure, a significant improvement in operational stability was observed for the devices using the DJ structure. This indicates that the chemical and crystal nature of 2D perovskites play a crucial role in determining the performance of PeLEDs.

To improve the structural properties of 2D perovskites even further, a novel approach with two different alternating cations in the interlayer space (ACI) has been introduced. ACI perovskites using guanidinium (GA) and methylammonium (MA) as the alternating spacer cations were first reported by Kanatzidis *et al*[20]. The ACI perovskites have an empirical chemical formula of (GA)(MA)_n_Pb_n_I_3n+1_. However, the unique ordering of the spacer cations makes their crystal structure reminiscent of that of DJ perovskites with a relative (1/2, 0) shift of the layers along the ab-plane, though the occupation of the interlayer sites is different due to charge balance restrictions[20]. Such a unique structure of ACI perovskites could lead to different photophysical and optoelectronic properties compared to the known 2D perovskites, especially considering that the replacement of the large cations with two different smaller cations will result in a much closer packing of the [Pb_n_I_3n+1_] slabs[20, 21]. Recently, by controlling the processing conditions of ACI perovskites, solar cells with power conversion efficiencies over 18% have been fabricated, demonstrating the potential of such 2D perovskites for high-performance photonic devices[21-23]. Nevertheless, to the best of our knowledge, the employment of ACI perovskites in PeLEDs has not been evaluated despite the beneficial properties that can be expected, e.g., (1) improved charge transport due to the use of smaller spacer cations and (2) an increased number of hydrogen bonds and consequently a stronger crystal lattice due to the use of GA cations[24-27].

Herein, we present the first demonstration of PeLEDs based on ACI perovskites. Various characterizations were first applied to confirm the formation of ACI perovskites and to investigate their film morphology, crystal structure, optical properties, and photoluminescence temperature evolution. In particular, synchrotron-based grazing incidence wide angle X-ray scattering (GIWAXS) measurements of thin films confirm the phase purity of the ACI perovskites. In addition, transient absorption spectroscopy (TAS) was used to obtain insight into the charge carrier dynamics[28-30] in the ACI perovskites prepared with different n values, showing efficient charge funneling for the ACI perovskite with n = 3. Therefore, PeLEDs with a maximum EQE of 3.4% were achieved by using the ACI perovskite with n = 3 in a typical LED structure of ITO/PolyTPD/perovskites/TPBi/LiF/Al, where PolyTPD and TPBi refer to poly[N,N'-bis(4-butylphenyl)-N,N'-bis(phenyl)-benzidine] and 2,2',2"-(1,3,5-benzinetriyl)-tris(1-phenyl-1-H-benzimidazole), respectively[8, 31, 32]. Devices based on ACI retain approximately 80% of their initial performance after operating at 20 mA/cm^2^ for 350 min, outperforming the operational stability of PeLEDs fabricated with the same device structure but different perovskites[8, 17, 33]. This demonstrates the initial promise of ACI perovskites for stable PeLED application.

**2 Results and discussion**

**2.1 Crystal structure, morphology, and optical properties of ACI perovskite thin films**

ACI perovskite films, i.e., (GA)(MA)_n_Pb_n_I_3n+1_ with n = 1, 2 and 3 layers (denoted ACI 1, ACI 2 and ACI 3), were fabricated using a one-step antisolvent method of a precursor solution with the dripping of toluene during spincoating. The as-cast films were then annealed at a low temperature (70 °C) for 5 min to form perovskite films[21, 31]. We first studied the crystal structure of the obtained ACI perovskite films using grazing-incidence X-ray diffraction (GIWAXS) measurements (Figure 1a-c) that have been extensively used in recent years to characterize 2D and 3D perovskites[20, 21, 23, 34-37]. All ACI films show diffuse diffraction rings with strong diffraction spots indicating a preferential orientation of the perovskite grains with respect to the substrate surface [38]. IIn order to determine lattice parameters of organized nanostructures in thin films and to investigate the crystal orientation relative to the substrate, three-dimensional structure indexing has been performed and overlayed [39]. Using the data obtained on single ACI (n = 1-3) crystals[20], the powder diffraction patterns for corresponding crystalline phases have been simulated and compared to the azimuthal integration of GIWAXS data of ACI films (Figure S1). All peaks which exceed 30% of maximum simulated intensity value in the powder diffraction have been identified in the GIWAXS patterns. The lattice parameters have been adjusted (Table S1) to match the prominent reflections in GIWAXS [40](Figure 1a-c). A 5% increase in the lattice parameter along c of axis is observed for all ACI films compared to corresponding single crystal data[20]. The scattering features located at q = 4.5 nm^-1^ and around 8 nm^-1^ for the ACI 1 film (Figure 1a) suggest the presence of an intermediate MAPbI_3_ perovskite phase [21]. The ACI 3 film (Figure 1c) exhibits more pronounced in-plane and out-of-plane Bragg spots suggesting higher crystalline order in which crystal domains are preferentially oriented along their b and c axes perpendicular to the substrate. In order to quantify the degree of domain orientation with respect to the substrate, the azimuthal distributions of the reflections from (002), (004), and (006) planes (ACI 1-3 films, respectively) have been analyzed (Figure S2). Referring to the (00l) planes, the Hermans’ orientation function <P(θ)> has been calculated which provides an alternative interpretation of crystal orientation in relative to the interpretation of the azimuthal distribution of (020/101) peak as proposed in literature[21, 34]. <P(θ)> can vary between -0.5 and 1. When all the crystals in the film are oriented with their c axes parallel to the out-of-plane direction (the reference direction in our case) <P(θ)> is 1, when all the c axes are perpendicular to the reference direction (i.e. in-plane direction) <P(θ)> is -0.5, and when the crystals are randomly oriented <P(θ)> is 0. As it can be seen from Figure S2b the highest degree of orientation is observed for ACI 3 film (<P(θ)> = 0.28), while ACI 2 film has a very poor orientation (<P(θ)> = 0.06), and ACI 1 film shows an intermediate degree of orientation (<P(θ)> = 0.14).

The morphology of the ACI perovskite films was characterized by atomic force microscopy (AFM) measurements (Figure 1d-f). The AFM images show that for all three samples, the crystal grains are close-packed, but the topography and surface roughness are different. More detailed topography is shown in Figure S3. Many high features can be observed in the ACI 1 films, indicating a rougher surface with a root-mean-square (RMS) roughness of 13.60 nm. The number of such high features decreases with increasing n, as does the surface roughness, i.e., 10.33 nm and 7.40 nm for ACI 2 and ACI 3, respectively.

Figure 2a shows the UV–vis absorption spectra of the ACI perovskite (GA)(MA)_n_PbnI_3n+1_ films with different n values. All three thin films exhibit a band edge absorption position close to that of the MAPbI_3_ bulk phase, which suggests the existence of the bulk phase[41]. The absorption intensity of the ACI films in the range of ≈400–800 nm slightly changes with the GA/MA ratio. Similar to RP perovskites, the intensity of the excitonic peaks decreases with decreasing GA/MA ratio in the ACI perovskites as a consequence of the decrease in the dielectric confinement[20, 33]. Beyond the fundamental exciton n = 1 at 1.9 eV (654 nm), where n is an integer, we also observed harmonics with integer n equal to 2 and 3 at 2 eV (623 nm), and 2.14 eV (580 nm), representing different quantum well (QWs) structures (detailed in Figure S4)[20, 22]. In addition to the excitonic peaks, the ACI films simultaneously exhibit other high-energy peaks as weak shoulders from the preceding lower-dimensional perovskites, suggesting that the ACI films are a mixture of perovskite QWs with different bandgaps [42]. The absorption edge and the excitonic peak position are redshifted in the ACI perovskites compared to RP perovskites. This is also reflected in the color of the (GA)(MA)_n_PbnI_3n+1_ compound, which changes from orange-red (n = 1) to brown (n = 2) to dark brown (n = 3) (Figure S5). According to the Kubelka-Munk theory, the band edges can be calculated as 2.05 eV, 1.99 eV and 1.84 eV for the three QWs (Figure S6), suggesting the influence of the different GA/MA ratios, i.e., perovskite dimensionality tuning, on the band structure of the materials[20].

For ACI 1, ACI 2 and ACI 3 photoluminescence (PL), the maximum emission peaks are located at 680 nm, 740 nm, and 770 nm, respectively (Figure 2b). Notably, the FWHM of the ACI 1 peak is greater than that of ACI 2 and ACI 3, attributed to the existence of multiple emitting species. A complete overview of the various excitation wavelengths and emitting species of all systems under study is represented in Figure S7 through their excitation-emission maps. The PL homogeneity of the ACI samples can be directly observed in Figure S7. Note that further increasing the GA concentration beyond that of ACI 1, without dimethyl sulfoxide (DMSO) in the precursor, and inappropriate addition of antisolvent can cause phase separation, see Figure S8[28]. Herein, the crystal growth and orientation of the 2D perovskites could be induced by the strong coordination molecule DMSO when it was used in the precursor solution, leading to a decreased trap state density and more efficient charge transport compared to the normal 2D perovskite film[36]. The carrier lifetimes of the different ACI films were studied by time-resolved PL (TRPL) measurements (Figure 2c). The luminescence lifetimes of ACI 1, ACI 2, and ACI 3 films are compiled in Table S2. The shortest lifetime was recorded for the ACI 3 film, indicating the presence of faster radiance recombination. Note that PL linewidth broadening has a direct consequence with carrier phonon interactions. Strong carrier-phonon interaction has been invoked to account for below-band-gap self-trap of exciton states and broad emissions in quasi-2D layer perovskites. Self-trapping of exciton becomes more prominent with a decreasing number of n, since the reduction of the dimensionality of a crystalline system lowers the deformation energy, facilitating self-trapping [43-45]. Therefore, we attribute that the ACI 1 contains more phonon assisted exciton recombination in addition to free exciton recombination which increases the average lifetime of ACI 1[40]. Finally, the environmental stability of the optimized ACI 3 film was evaluated (Figure 2d), showing a marginal PL intensity change after being stored in air for 3 months in the dark as expected for such systems.

To obtain deeper insight into the photophysical properties of the systems under study, we investigated the temperature evolution of their PL between 300 and 4.2 K. PL spectra have been collected at an increment of 5K for all materials. PL spectra at selected temperatures are depicted in Figure 3a (PL spectra for all temperatures are shown in Figure S9). For ACI 1 and 2, several PL peaks are observed at lower temperatures whose origin is under debate and has been attributed to the presence of multiexciton correlations (many-body electron-hole correlations), free and bound excitons as well as bi‐excitons or coexistence of different phases of 2D perovskites[46]. ACI 3 however, shows two peaks down to 4.2 K. Note that for all three systems, we considered the two peaks present at room temperature for further analysis (shown by arrows in Figure 3a and annotated as p#1 and p#2 in Figure 3b-e). An example of the fitting procedure and the two Gaussian peaks extracted for all materials at room temperature is depicted in Figure S10. The temperature dependence of the two PL peak centers is depicted in Figure 3b, exhibiting approximately the same behavior for all three systems, with no abrupt changes and hence no structural phase transitions.

The exciton binding energy of all systems under study was estimated by applying the Arrhenius equation to the temperature dependence of their PL integrated intensity, as illustrated in Figure 3(c-e):

*I*(𝑇) = 𝐼_0_/ (1 + 𝐴𝑒 ^−𝐸^_𝑏_^/𝑘^_𝐵_^𝑇^)

where I_0_ and A are constants, kB is the Boltzmann constant, and Eb is the exciton binding energy. The solid curves are the fits. The exciton binding energy for ACI 3 decreases drastically (Eb = 110 meV) compared to those of ACI 1 and ACI 2 (on the order of 200-300 meV) due to the reduced quantum confinement effect in ACI 3 [28, 47]. It is noteworthy that the exciton binding energy of ACI 3 is still much larger than that of common MAPbI3 perovskites [48, 49]. Observing such a low exciton binding energy for ACI 3 hints at a transition from a confined 2D system (i.e., ACI 1 and 2) to a bulk system when varying the number of layers in the structure. Furthermore, the fact that more peaks are observed for ACI 1 and 2 at lower temperatures points towards their more confined structures where many-body electron-hole correlations are more prominent compared to ACI 3[46].

**2.2 TAS study on the obtained ACI perovskites**

Next, to gain further insight into the charge carrier dynamics of the perovskites with different numbers of layers, we performed femtosecond TAS. In these experiments, a fs pulse (pump) was used to excite the perovskite films. The changes in absorption (ΔA) were probed by a second fs pulse (probe), and the spectra were recorded at a certain delay between the two pulses. Figure 4a and Figure S11 show the fs TA spectra obtained for ACI 1, 2, and 3 thin films. The corresponding bleaching kinetics of ACI 3 at 530 nm, 580 nm, 620 nm and 730 nm in a 50 ps time window are illustrated in Figure 4b-e. In these experiments, a lock-in technique (heterodyne) was used, and the obtained spectra were fitted with a multiexponential function[50-52]. All fs TA spectra are characterized by a positive and long-lived band in the wavelength range of 500-580 nm, which is formed with a time constant of 300 fs and indicates photoinduced absorption due to refractive index changes and exciton generation[53]. The band decays with time constants of ~4.1 ps and greater than 50 ps attributed to fast exciton relaxation and dissociation (Figure 4b), respectively. The TA spectra also feature strong negative bands related to different QWs at 580-590 (n = 1), 600-620 (n = 2) and 640-660 nm (n = 3), assigned to the maximum valence band (VB) depopulation[22, 23]. The spectral positions of the photoinduced bleaching (PIB) bands correspond to the excitonic peaks observed in stationary absorption and provide information about the carrier dynamics and density. These noteworthy signals indicate that for n = 1, the intense PIB occurs rapidly (time constant of 130 fs at 580 nm), while for n = 2, this occurs slightly slower (time constant of 500 fs at 620 nm). This phenomenon is due to the dielectric confinement being reduced for the n > 1 systems; hence, the exciton formation time is slower (Figure 4c-d). For n > 3, the exciton formation slows down further (time constant of 810 fs), as this band at 740 nm reaches a maximum in 3 ps (Figure 4e). These bands indicate depopulation with time constants of 4-30 ps and can be tentatively attributed to exciton relaxation in the conduction band (Figure 4a, c, d). This is also supported by the bathochromic shift of the bands (Figure 4a and S6, *e.g.,* the n =1 band shifts from 580 to ≈590 nm).

Earlier reports on the vertically stacked microcrystals of low-dimensional ACI perovskites assembled within 3D perovskite nanoscale networks attributed such prompt time constants to charge carrier localization from low-dimensional ACI perovskites to 3D perovskites (with a smaller bandgap)[23]. According to our data, these band depopulation decay time constants are on the order of ps or longer. For instance, when comparing the TA dynamics of ACI 3 with the ACI 1 and ACI 2, the band attributed to n = 1 in sample ACI 1 decay much slower than in sample ACI 3 due to a reduced presence of QW with other n-values (Figure 4a, Figure S11). Interestingly, the TA spectra of ACI 3 feature additional bands located at 670 nm and higher wavelengths, indicating exciton resonances of ACI perovskites with n>3. The formation of these species is even slower than that for n=3 but faster than their decay. Thus, energy transfer possibly occurs towards the structural phases with a higher number of layers (possibly bulk 3D). In contrast to ACI 1 and ACI 2, the TA spectra of ACI 3 show an intense photobleaching (PB) signal in the 700-730 nm region, whose origin can be assigned to transitions between the VB and conduction band[53]. These results clearly indicate that a substantial portion of the photogenerated excitons from lower n QWs (high bandgap) will be gradually localized to higher n QWs (low bandgap) in the mixed ACI samples. In addition, this observed charge carrier localization in ACI perovskites is slightly faster than that in previously reported 2D RP perovskites[50-52]. The faster charge carrier localization may be caused by the shortened distance between the inorganic layers and the optimized special arrangement of the cations in the ACI perovskite.

**2.3 Performance of PeLEDs fabricated with ACI perovskites**

Finally, we evaluated the performance of the different ACI films in PeLEDs. The devices were prepared with a conventional architecture of ITO/PolyTPD/perovskite/TPBi/LiF/Al (Figure 5, together with the fabrication process[54, 55]). Figure 6a presents the typical electroluminescence (EL) spectra recorded from the devices made with ACI 1, ACI 2 and ACI 3, which resemble the PL spectra shown in Figure 2b. Figure 6b presents the radiance-voltage curves of the corresponding devices, showing similar turn-on voltages of ~2.1 V. The turn-on voltages are very similar for the devices employing the same device structure with PolyTPD and TPBi as the hole and electron injection layers. The radiance value increases in the sequence of ACI 1, ACI 2 and ACI 3. It reaches 77.49 W Sr^−1^ m^−2^ at 6 V for ACI 3, outperforming those of ACI 1 (1.15 W Sr^−1^ m^−2^) and ACI 2 (31.82 W Sr^−1^ m^−2^) at the same voltage. The radiance increases sharply once the voltage reaches the threshold voltage, demonstrating a low series resistance and efficient carrier injection into the device[19]. According to the EQE-current density (EQE-J) curves, the ACI 3 device achieves a maximum EQE of 3.4% at 249 mA cm^−2^, while the maximum EQEs of the ACI 1 and ACI 2 devices are 1.56% and 0.11%, respectively. The relatively high EQE can be maintained in a wide range of current densities, e.g., it remains above 2.5%for J up to 720 mA cm^−2^ (Figure 6c). The low efficiency roll-off is superior to most of the reported perovskite LED devices based on hybrid perovskite structure[56]. The better device performance of ACI 3 agrees with its shorter carrier lifetime, more uniform film, higher PL quantum yield (PLQY), more efficient carrier funneling, and higher exciton binding energy than typical 3D perovskites. More encouragingly, the ACI 3 device maintains over 80% of its initial performance after operating at 20 mA/cm^2^ for 350 min (Figure 6d), outperforming devices based on other types of perovskites with the same device structure[7, 8, 17, 33, 57]. In Table S3, we summarize the EL peak wavelength, maximum EQE, operation stability and test conditions at a constant current density of the thin film LEDs in this work as well as other thin films in previous literature. We note that the emission spectrum also remains the same before and after bias application, indicating the excellent phase stability (Figure S12). Therefore, we believe that ACI perovskites with further optimized compositions are promising candidates to achieve highly stable and efficient PeLEDs.

**3 Conclusion**

In summary, 2D ACI perovskites have been applied in PeLEDs as the active layer for the first time. The morphological and optoelectronic properties of the ACI perovskites were investigated for varying n between 1 and 3. Our results indicate that the ACI 3 perovskite film has favorable characteristics for PeLEDs. This is confirmed by the PeLED device performance. We achieve the highest EQE of 3.4% for devices using ACI 3 perovskites. Such devices also show encouraging operational stability, maintaining 80% of the initial performance and without changes in the emission spectrum after being biased at 20 mA/cm^2^ for 350 min. Our results demonstrate that 2D ACI perovskites are an alternative platform for further investigation to achieve high-efficiency and stable PeLEDs.

**4 Experimental section**

**4.1 Device structure**

The near-infrared (NIR) emission LEDs were made with a device structure of ITO/poly[N,N′-bis(4-butylphenyl)-N,N-bis(phenyl)-benzidine] (PolyTPD)/perovskite/1,3,5-tris(1-phenyl-1H-benzimidazol-2-yl)benzene (TPBi)/LiF/Al (Figure 2a).

**4.2 Materials**

Lead iodide (PbI_2_, 99%) powder, methylammonium iodide (MAI, 99%) powder, guanidine iodide (GAI, 99%) powder, toluene (anhydrous, 99.8%), N,N-dimethylformamide (DMF, anhydrous, 99.8%) and dimethyl sulfoxide (DMSO, anhydrous, 99.8%) were purchased from Alfa Aesar. LiF powder was obtained from Sigma-Aldrich. PolyTPD and TPBi were purchased from American Dye Source and Lumtec, respectively. All of the commercial materials were used as received.

**4.3 Material synthesis and film preparation**

Hybrid ACI 2D perovskite (GA)(MA)_n_Pb_3n+1_ films were fabricated using one-step spin coating with antisolvent (chlorobenzene) dripping from the precursor solution. For ACI perovskites with pure iodide, GAI and MAI in different molar ratios (1~0.3:1) were dissolved together with PbI_2_ (461 mg, 1 mmol) in a mixture solution of DMF and DMSO (volume ratio, 9:1) under magnetic stirring for 24 h to obtain a 0.4 M GAMA_n_Pb_n_I_3n+1_ (n=1, 2, 3) precursor solution, which was used to deposit NIR emissive ACI perovskite films. The perovskite films were deposited by spin coating at 4000 rpm. An antisolvent step was performed by dropping toluene on the substrates 5 s after commencing spinning. The samples were then annealed at 70 °C for 10 min.

**4.4 Femtosecond TA experiments**

An amplified femtosecond double optical parametric amplifier (OPA) laser system was used to generate the excitation and probe pulses. The assembly of a Ti-sapphire oscillator (Mai Tai SP, Spectra-Physics) coupled to a laser amplifier comprising a stretcher, a Ti-sapphire regenerative cavity amplifier and a compressor (Spitfire Pro 35F-XP, Spectra-Physics) was the source of 35 fs pulses (FWHM). The pulses had a bandwidth of 31 nm (FWHM), a 4 mJ pulse energy around 800 nm and a repetition rate of 1 kHz. The regenerative amplifier was energized by a Q-switched, diode-pumped Nd: YLF pulsed laser (Empower-30, Spectra-Physics) capable of delivering a 527 nm output beam of 30 watts. The amplifier output was used to pump two identical two-stage OPAs of white-light continuum (Topas-C, Light Conversion). Each OPA was pumped with a 1 mJ pulse energy producing 60 fs (FWHM), 31 nm bandwidth (FWHM) pulses. The energy of the independently tunable excitation pulses was in the range of 100 μJ @ 500 nm, and the output could be tuned over a spectral domain from 300 to 2600 nm. A small percentage of the regenerative amplifier output was used to generate a white-light continuum that then served as probe light. This was done by focusing a small part of the 800 nm beam into a 3 mm sapphire plate to obtain white light in the 450-800 nm region. Monochromatic detection was performed using a photomultiplier tube (PMT) (Hamamatsu R928), whereas the spectra were recorded using a charge-coupled device (CCD) camera (Princeton Instrument Pixis 100) mounted at the exit ports of a 300 mm focal length spectrograph (Acton Research 2300). The entire system provides pulses with a duration of 100 fs (FWHM cross correlation between pump and probe) at a repetition rate of 1 kHz. After each experiment, the integrity of the samples was checked by recording the steady-state absorption and emission spectra.

**4.5 Device fabrication**

PolyTPD was spincoated on precleaned ITO (Colorado Concept Coatings) at 1000 rpm for 60 s, followed by thermal annealing at 150 °C for 20 min. To improve the surface wettability, the PolyTPD layer was treated with O_2_ plasma for 6 s at a power of 100 W. After that, the perovskite films were deposited in a N_2_-filled glove box as described in the perovskite film preparation section. Then, TPBi, LiF, and Al layers were thermally evaporated on top of the perovskite films sequentially, with thicknesses of 60, 1.2, and 100 nm, respectively. The device area was 0.1 cm^2^ with dimensions of 2 × 5 mm.

**4.6 Characterizations**

GIWAXS measurements have been performed at the Austrian SAXS beamline at the ELETTRA synchrotron in Trieste (Italy) at a photon energy of 8 keV[58]. The beam size was set to 1x0.1 mm^2^ (HxV) while the sample to detector (Pilatus3 1M, Dectris) distance was adjusted to 217.3 mm using a silver behenate as a reference pattern. All measurements have been performed in air at the incident angle of 0.5º. The patterns have been corrected for the fluctuations of the primary intensity[59]. The simulations of the GIWAXS patterns have been performed with the GIXSGUI toolbox[39] that has also been used for extraction of the azimuthal intensity profiles in equal Δq interval both in the proximity of (002), (004), and (006) reflections for ACI 1-3 films at lower q to be used for background estimation, respectively. The successive background subtraction from the azimuthal peak intensity profiles and calculation of the Hermans’ orientation function <P(θ)> [60] has been performed using IGOR Pro (IGOR Pro 7.0.8.1, WaveMetrics). The powder diffraction patterns have been simulated with Diamond 3.2i (Crystal Impact). AFM measurements were performed in air using a Multimode AFM with a Nanoscope VIII controller (Bruker) in tapping mode. Olympus silicon cantilevers (AC160TS-R3) with a nominal frequency resonance of 300 kHz and spring constant around 26 N m-1 were employed. The value for the RMS roughness for each sample consists of an average obtained from 16 different areas of 500 nm x 500 nm. Gwyddion software was used for AFM image processing (Nečas, D.; Klapetek, P. Gwyddion: An Open-Source Software for SPM Data Analysis. Central European Journal of Physics. January 1, 2012, pp 181–188). UV–vis diffuse reflectance spectra (UV-vis DRS) were measured using a Lambda 950 UV–vis spectrophotometer. PL measurements were performed on an Edinburgh FLS980 using a He-Cd laser with a 470 nm excitation wavelength at room temperature. Lifetime spectra were measured using Leica TCS SP8 X ‘FLIM’ measurements with a 470 nm laser at a 10 MHz repetition rate. Temperature-dependent PL experiments were performed using a Thorlabs M365LP1 solid-state laser with a DC2200 driver operating at 365 nm. The sample was excited via a 550 μm core optical fiber, and the PL spectra were collected through 11 core optical fibers of 200 μm diameter that surrounded the excitation fiber. The detection fibers were coupled to a LOT-QD Shamrock F/4 spectrometer with an iXon DV887 electron multiplying CCD (EMCCD). The spectra were collected every 5 K between 4.2 and 300 K in a He flow cryostat.

**Acknowledgments:** B.P., M.K. and E.D acknowledge support from the Research Foundation-Flanders through postdoctoral fellowships (FWO Grants No. 1275521N, 12Y6418N and 12O3719N, respectively as well FWO project G098319N to M.K. and “Krediet aan Navorsers” 1514220N)). J.H and M.B.J.R acknowledge financial support from the Research Foundation- (FWO) through research projects (FWO Grant No’s G098319N and ZW15_09-GOH6316), from the Flemish government through long term structural funding Methusalem (CASAS2, Meth/15/04) and through iBOF funding (PERsist: iBOF-21-085) and from the KU Leuven Research Fund (grants C14/15/053 and C14/19/079). M.B.J.R acknowledges financial support from the KU Leuven Industrial Research Fund (C3/19/046). Y. Z. acknowledge support from the China Scholarship Council (No. 201908440479). P. H. acknowledges funding from the European Research Council under the European Horizon 2020 Programme/ERC grant agreement no. 835133 (ULTRA-LUX). This project received funding from the EU-H2020 Research and Innovation Programme under grant agreement no. 654360 having benefitted from access to the Austrian SAXS beamline provided by TUG@Elettra in Trieste, Italy, within the framework of the NFFA-Europe Transnational Access Activity.

**Author contribution:** Y. Z. conducted most of the experiments and their analysis, and wrote the manuscript under the supervision of J.H., W.Q. and B.P., M.K. and J.V.V. assisted with the low temperature PL experiments and analysis., W.Q. prepared and characterized the LED devices under supervision of P.H., D.N. and H.A. assisted with the GIWAXS experiments and analysis., and E.F. performed the femtosecond TAS characterizations. M.C.R.G performed the AFM measurement under supervision of S.D.F. The research line presented in the manuscript was conceived by J.H., B.P., M.B.J.R. and E.D. All the authors contributed to the writing and correcting of the manuscript, have accepted responsibility for the entire content of this submitted manuscript and approved submission.

**Conflict of interest statement:** The authors declare no conflicts of interest regarding this article.


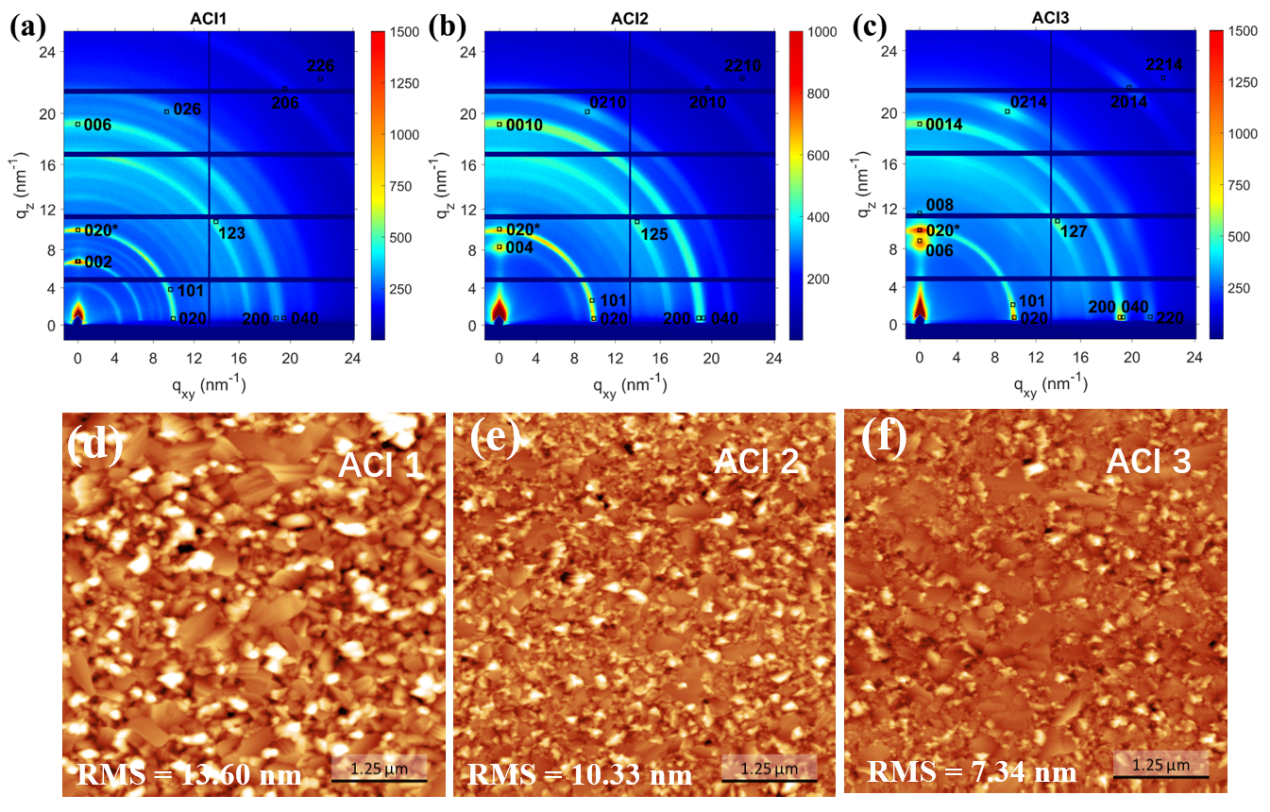


**Figure 1.** a-c) 2D GIWAXS patterns of (GA)(MA)_n_Pb_n_I_3n+1_ (ACI, n = 1-3) films acquired at the incidence angle of 0.5°. The reflections are indexed according to the adjusted unit cell parameters given in Table S1. d-f) AFM images (5 μm x 5 μm) and RMS roughness of the ACI films (Roughness with standard deviation, ACI 1: 13.60 ± 5.53 nm, ACI 2: 10.33 ± 2.46 nm, ACI 3: 7.34 ± 1.98 nm).


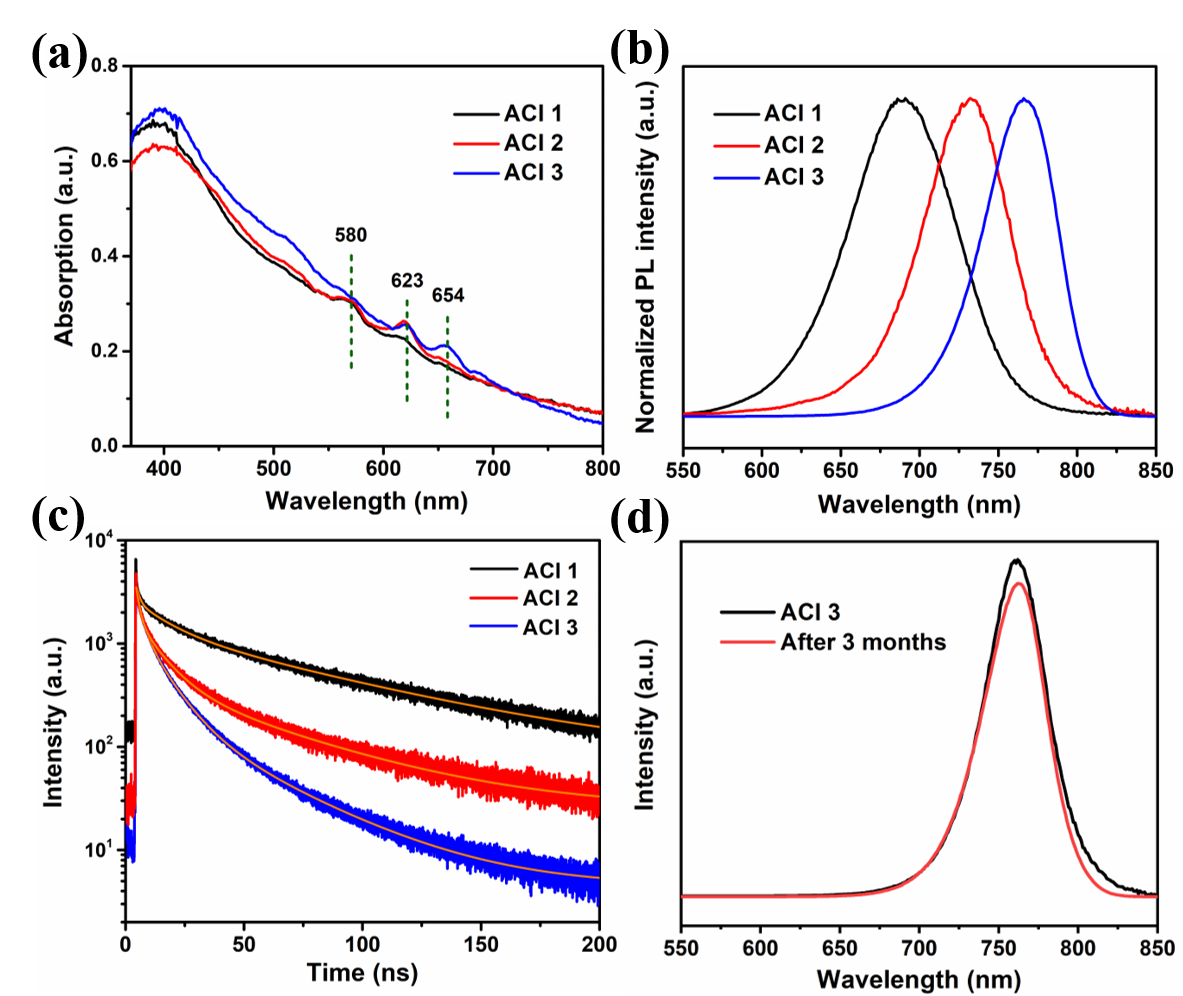


**Figure 2.** a) UV–vis absorption spectra, b) steady-state PL spectra excited at 470 nm, and c) TRPL spectra of the ACI (n = 1, 2, 3) perovskite films. d) PL spectra of ACI 3 films measured at Day 0 and Day 90 when stored in air.





**Figure 3.** Temperature evolution of the PL properties of ACI 1, 2 and 3. a) PL spectra at different temperatures for ACI 1, 2 and 3. The two peaks (annotated as p#1 and p#2) indicated by arrows for n = 1, 2 and for n = 3 are considered for further analysis. b) Temperature dependence of the PL peak centers of the peaks indicated by arrows in (a) for all three materials. c-e) PL integrated intensity as a function of temperature (*T*) for ACI 1 (c), ACI 2 (d) and ACI 3 (e). The exciton binding energies are extracted using the Arrhenius equation shown by solid lines in Figure (c-e).


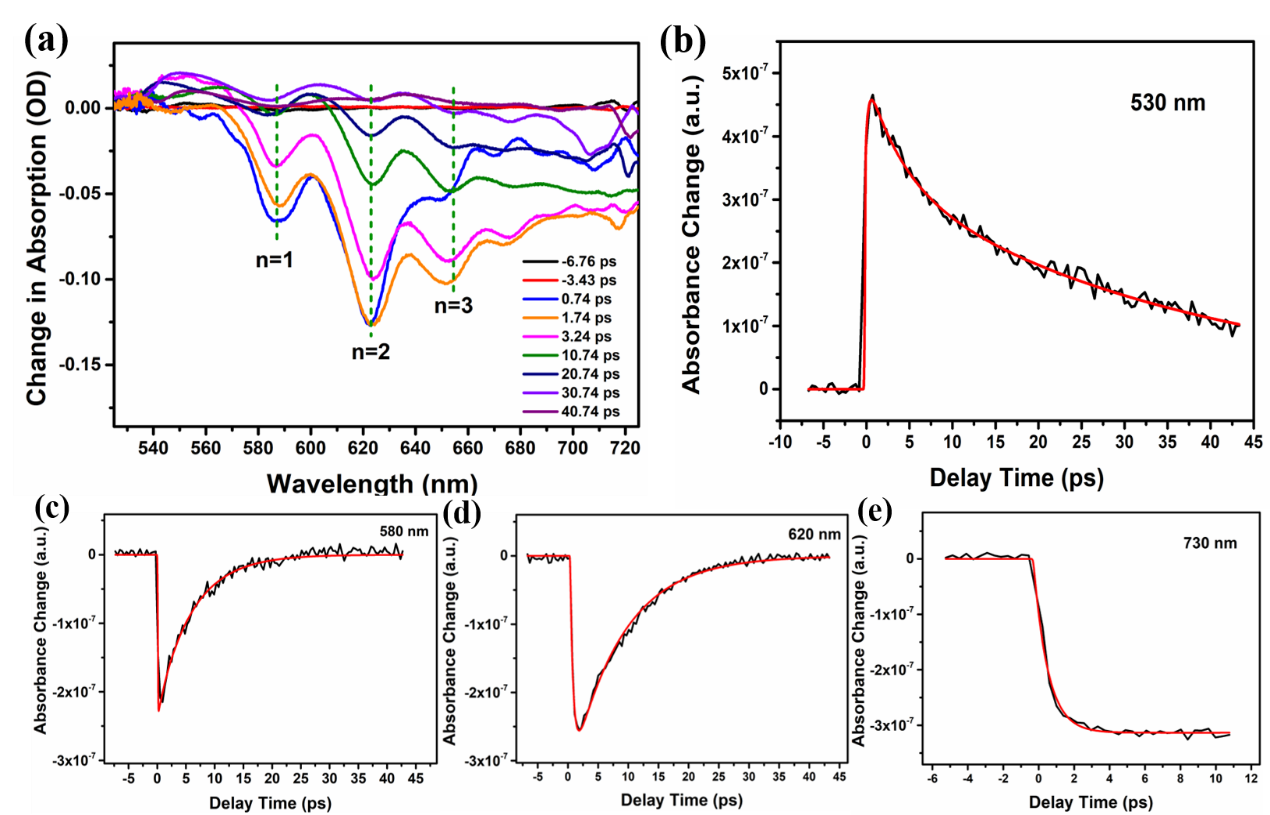


**Figure 4.** a) TA spectra obtained for thin film ACI 3 in a 50 ps time window. b-e) TA decay traces and corresponding fits obtained for thin film ACI 3 at 530 nm (b), 580 nm (c), 620 nm (d) and 730 nm (e) in a 50 ps time window.


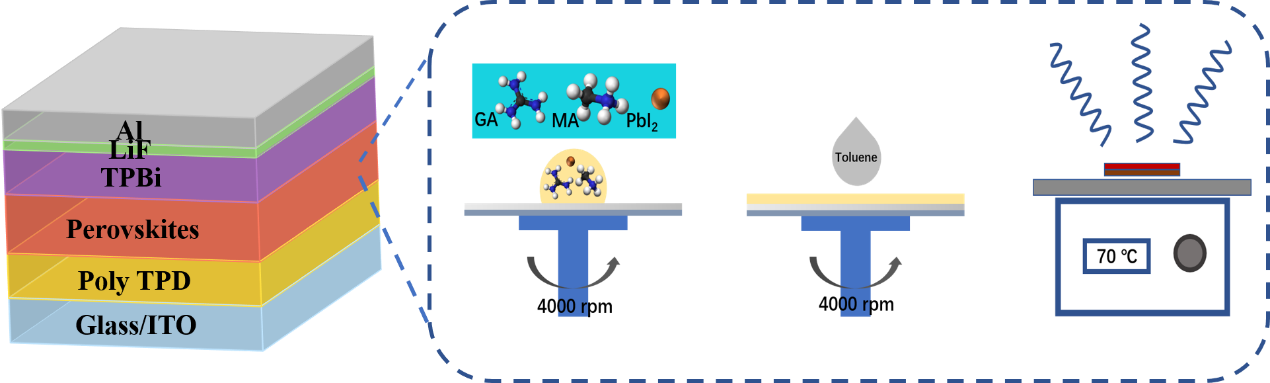


**Figure 5.** Schematic of the perovskite LED structure used in this work, along with the perovskite structure and fabrication process. The perovskite films were deposited by spin coating at 4000 rpm. An antisolvent step was performed by dropping toluene on the substrates 5 s after commencing spinning. The samples were then annealed at 70 °C for 10 min.


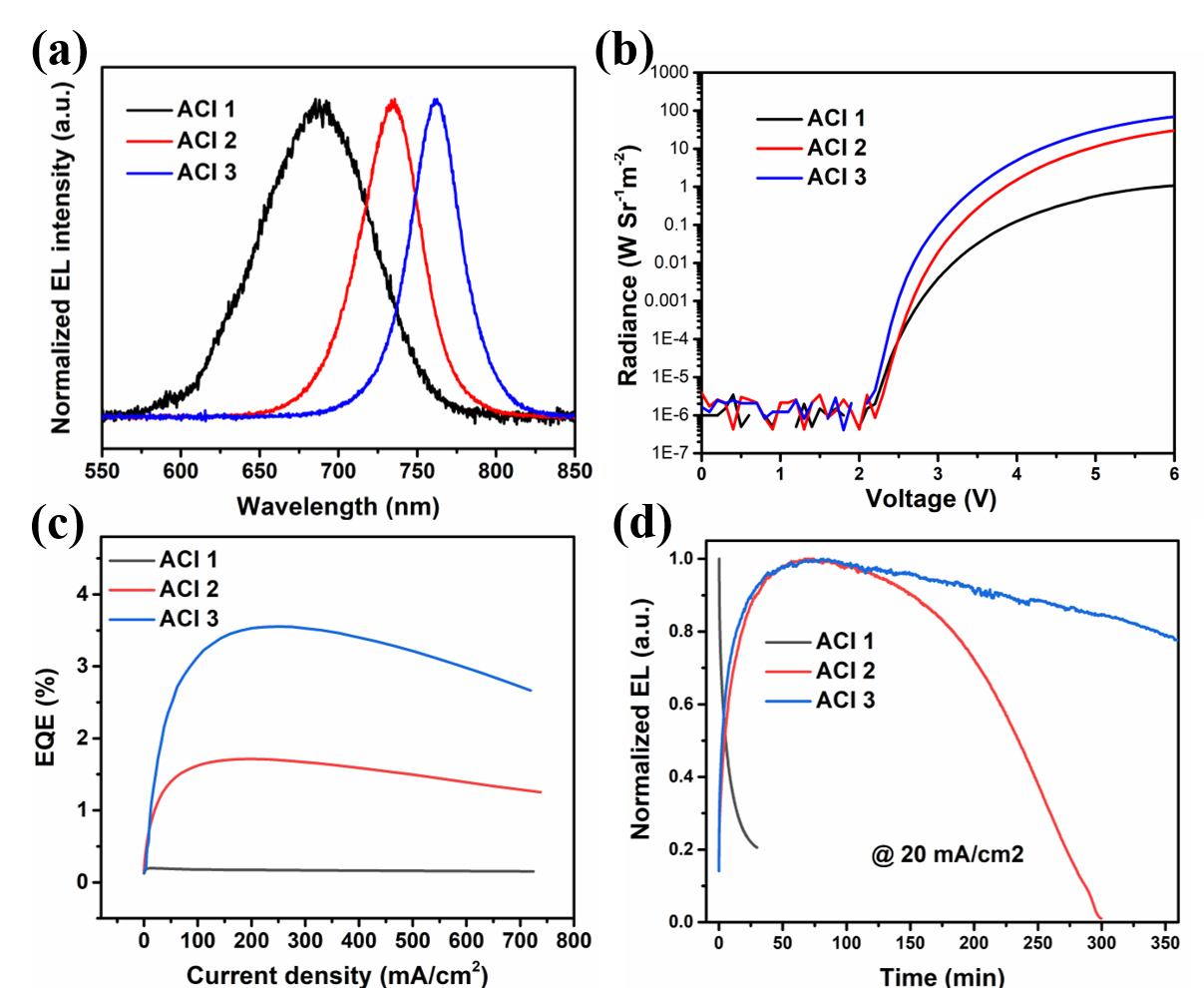


**Figure 6.** a) EL spectra of the ACI perovskites from the corresponding devices. b) Radiance versus voltage curves of LEDs based on ACI films. c) EQE versus current density characteristics of PeLEDs based on ACI films. d) Normalized EL intensity of device emitting layers under a constant current density of 20 mA cm^−2^.

[1] G. Grancini, and M. K. Nazeeruddin, "Dimensional tailoring of hybrid perovskites for photovoltaics," *Nat. Rev. Mater.*, vol. 4, pp. 4-22, 2018.

[2] L. Mao, W. Ke, L. Pedesseau et al., "Hybrid Dion-Jacobson 2D Lead Iodide Perovskites," *J. Am. Chem. Soc.*, vol. 140, pp. 3775-3783, 2018.

[3] G. Kieslich, S. Sun, and A. K. Cheetham, "Solid-state principles applied to organic–inorganic perovskites: new tricks for an old dog," *Chem. Sci.*, vol. 5, pp. 4712-4715, 2014.

[4] H. Tsai, W. Nie, J. C. Blancon et al., "High-efficiency two-dimensional Ruddlesden-Popper perovskite solar cells," *Nature*, vol. 536, pp. 312-316, 2016.

[5] C. Liang, H. Gu, Y. Xia et al., "Two-dimensional Ruddlesden–Popper layered perovskite solar cells based on phase-pure thin films," *Nat.Energy*, vol. 6, pp. 38-45, 2020.

[6] K. Leng, I. Abdelwahab, I. Verzhbitskiy et al., "Molecularly thin two-dimensional hybrid perovskites with tunable optoelectronic properties due to reversible surface relaxation," *Nat. Mater.*, vol. 17, pp. 908-914, 2018.

[7] Y. Chen, Y. Sun, J. Peng et al., "2D Ruddlesden-Popper Perovskites for Optoelectronics," *Adv. Mater.*, vol. 30, pp. 1703487, 2018.

[8] L. Zhao, K. M. Lee, K. Roh et al., "Improved Outcoupling Efficiency and Stability of Perovskite Light-Emitting Diodes using Thin Emitting Layers," *Adv. Mater.*, vol. 31, pp. e1805836, 2019.

[9] S. Yang, W. Niu, A.-L. Wang et al., "Ultrathin Two-Dimensional Organic-Inorganic Hybrid Perovskite Nanosheets with Bright, Tunable Photoluminescence and High Stability," *Angew. Chem. Int. Ed. Engl.*, vol. 56, pp. 4252-4255, 2017.

[10] X. Li, W. Ke, B. Traore et al., "Two-Dimensional Dion-Jacobson Hybrid Lead Iodide Perovskites with Aromatic Diammonium Cations," *J. Am. Chem. Soc.*, vol. 141, pp. 12880-12890, 2019.

[11] C. C. Stoumpos, D. H. Cao, D. J. Clark et al., "Ruddlesden–Popper Hybrid Lead Iodide Perovskite 2D Homologous Semiconductors," *Chem. Mater.*, vol. 28, pp. 2852-2867, 2016.

[12] H. Lin, C. Zhou, Y. Tian et al., "Low-Dimensional Organometal Halide Perovskites," *ACS Energy Lett.*, vol. 3, pp. 54-62, 2017.

[13] Y. Cao, N. Wang, H. Tian et al., "Perovskite light-emitting diodes based on spontaneously formed submicrometre-scale structures," *Nature*, vol. 562, pp. 249-253, 2018.

[14] R. Gautier, F. Massuyeau, G. Galnon et al., "Lead Halide Post-Perovskite-Type Chains for High-Efficiency White-Light Emission," *Adv. Mater.*, vol. 31, pp. e1807383, 2019.

[15] Y. H. Kim, H. Cho, and T. W. Lee, "Metal halide perovskite light emitters," *Proc. Natl. Acad. Sci. U.S.A.*, vol. 113, pp. 11694-11702, 2016.

[16] Z. K. Tan, R. S. Moghaddam, M. L. Lai et al., "Bright light-emitting diodes based on organometal halide perovskite," *Nat. Nanotechnol.*, vol. 9, pp. 687-692, 2014.

[17] Z. Xiao, R. A. Kerner, L. Zhao et al., "Efficient perovskite light-emitting diodes featuring nanometre-sized crystallites," *Nat. Photonics*, vol. 11, pp. 108-115, 2017.

[18] P. Cheng, Z. Xu, J. Li et al., "Highly Efficient Ruddlesden–Popper Halide Perovskite PA2MA4Pb5I16 Solar Cells," *ACS Energy Lett.*, vol. 3, pp. 1975-1982, 2018.

[19] Y. L. Yuequn Shang, Qi Wei et al., "Highly stable hybrid perovskite light-emitting diodes based on Dion-Jacobson structure," *Sci. Adv.*, vol. 5, pp. eaaw8072, 2019.

[20] C. M. M. Soe, C. C. Stoumpos, M. Kepenekian et al., "New Type of 2D Perovskites with Alternating Cations in the Interlayer Space, (C(NH2)3)(CH3NH3)nPbnI3n+1: Structure, Properties, and Photovoltaic Performance," *J. Am. Chem. Soc.*, vol. 139, pp. 16297-16309, 2017.

[21] Y. Zhang, P. Wang, M.-C. Tang et al., "Dynamical Transformation of Two-Dimensional Perovskites with Alternating Cations in the Interlayer Space for High-Performance Photovoltaics," *J. Am. Chem. Soc.*, vol. 141, pp. 2684-2694, 2019.

[22] T. Luo, Y. Zhang, Z. Xu et al., "Compositional Control in 2D Perovskites with Alternating Cations in the Interlayer Space for Photovoltaics with Efficiency over 18%," *Adv. Mater.*, vol. 31, pp. 1903848, 2019.

[23] H. Gu, C. Liang, Y. Xia et al., "Nanoscale hybrid multidimensional perovskites with alternating cations for high performance photovoltaic," *Nano Energy*, vol. 65, pp. 104050, 2019.

[24] S. Kanno, Y. Imamura, and M. Hada, "First-Principles Calculations of the Rotational Motion and Hydrogen Bond Capability of Large Organic Cations in Hybrid Perovskites," *J. Phys. Chem. C.*, vol. 122, pp. 15966-15972, 2018.

[25] G. Giorgi, and K. Yamashita, "Zero-dipole molecular organic cations in mixed organic-inorganic halide perovskites: possible chemical solution for the reported anomalous hysteresis in the current-voltage curve measurements," *Nanotech.*, vol. 26, pp. 442001, 2015.

[26] G. Giorgi, J.-I. Fujisawa, H. Segawa et al., "Organic–Inorganic Hybrid Lead Iodide Perovskite Featuring Zero Dipole Moment Guanidinium Cations: A Theoretical Analysis," *J. Phys. Chem. C.*, vol. 119, pp. 4694-4701, 2015.

[27] N. De Marco, H. Zhou, Q. Chen et al., "Guanidinium: A Route to Enhanced Carrier Lifetime and Open-Circuit Voltage in Hybrid Perovskite Solar Cells," *Nano Lett.*, vol. 16, pp. 1009-1016, 2016.

[28] M. Yuan, L. N. Quan, R. Comin et al., "Perovskite energy funnels for efficient light-emitting diodes," *Nat. Nanotechnol.*, vol. 11, pp. 872-877, 2016.

[29] J. Liu, J. Leng, K. Wu et al., "Observation of Internal Photoinduced Electron and Hole Separation in Hybrid Two-Dimentional Perovskite Films," *J. Am. Chem. Soc.*, vol. 139, pp. 1432-1435, 2017.

[30] Y. Fu, W. Zheng, X. Wang et al., "Multicolor Heterostructures of Two-Dimensional Layered Halide Perovskites that Show Interlayer Energy Transfer," *J. Am. Chem. Soc.*, vol. 140, pp. 15675-15683, 2018.

[31] W. Qiu, Z. Xiao, K. Roh et al., "Mixed Lead-Tin Halide Perovskites for Efficient and Wavelength‐Tunable Near-Infrared Light-Emitting Diodes," *Adv. Mater.*, vol. 31, pp. 1806105, 2018.

[32] J. H. Warby, B. Wenger, A. J. Ramadan et al., "Revealing Factors Influencing the Operational Stability of Perovskite Light-Emitting Diodes," *ACS Nano*, vol. 14, pp. 8855-8865, 2020.

[33] Z. Wang, F. Wang, B. Zhao et al., "Efficient Two-Dimensional Tin Halide Perovskite Light-Emitting Diodes via a Spacer Cation Substitution Strategy," *J. Phys. Chem. Lett.*, vol. 11, pp. 1120-1127, 2020.

[34] L. Oesinghaus, J. Schlipf, N. Giesbrecht et al., "Toward Tailored Film Morphologies: The Origin of Crystal Orientation in Hybrid Perovskite Thin Films," *Adv. Mater. Interfaces*, vol. 3, pp. 1600403, 2016.

[35] R. Yang, R. Li, Y. Cao et al., "Oriented Quasi-2D Perovskites for High Performance Optoelectronic Devices," *Adv. Mater.*, vol. 30, pp. e1804771, 2018.

[36] D. Lu, G. Lv, Z. Xu et al., "Thiophene-Based Two-Dimensional Dion-Jacobson Perovskite Solar Cells with over 15% Efficiency," *J. Am. Chem. Soc.*, vol. 142, pp. 11114-11122, 2020.

[37] F. Krieg, P. C. Sercel, M. Burian et al., "Monodisperse Long-Chain Sulfobetaine-Capped CsPbBr3 Nanocrystals and Their Superfluorescent Assemblies," *ACS Cent. Sci.*, vol. 7, pp. 135-144, 2021.

[38] E. S. Julian A. Steele, Handong Jin et al., "Texture Formation in Polycrystalline Thin Films of All-Inorganic Lead Halide Perovskite," *Adv. Mater.*, vol. 33, pp. 2007224, 2021.

[39] Z. Jiang, "GIXSGUI: a MATLAB toolbox for grazing-incidence X-ray scattering data visualization and reduction, and indexing of buried three-dimensional periodic nanostructured films," *J. Appl. Crystallogr.*, vol. 48, pp. 917-926, 2015.

[40] S. Ghosh, B. Pradhan, Y. Zhang et al., "Investigation of Many-Body Exciton Recombination and Optical Anisotropy in Two-Dimensional Perovskites Having Different Layers with Alternating Cations in the Interlayer Space," *J. Phys. Chem. C.*, vol. pp. 2021. (DOI: 10. 1021/acs. jpcc. 1c01318)

[41] V. A. Dong Shi, Riccardo Comin,et al., "Low trap-state density and long carrier diffusion in organolead trihalide perovskite single crystals," *Science*, vol. 347, pp. 519-522, 2015.

[42] J. Xu, O. Voznyy, M. Liu et al., "2D matrix engineering for homogeneous quantum dot coupling in photovoltaic solids," *Nat. Nanotechnol.*, vol. 13, pp. 456-462, 2018.

[43] X. Wu, M. T. Trinh, D. Niesner et al., "Trap states in lead iodide perovskites," *J. Am. Chem. Soc.*, vol. 137, pp. 2089-2096, 2015.

[44] M. D. Smith, A. Jaffe, E. R. Dohner et al., "Structural origins of broadband emission from layered Pb-Br hybrid perovskites," *Chem. Sci.*, vol. 8, pp. 4497-4504, 2017.

[45] J. Li, J. Ma, X. Cheng et al., "Anisotropy of Excitons in Two-Dimensional Perovskite Crystals," *ACS Nano*, vol. 14, pp. 2156-2161, 2020.

[46] E. P. Booker, M. B. Price, P. J. Budden et al., "Vertical Cavity Biexciton Lasing in 2D Dodecylammonium Lead Iodide Perovskites," *Adv. Opt. Mater.*, vol. 6, pp. 1800616, 2018.

[47] L. Pedesseau, D. Sapori, B. Traore et al., "Advances and Promises of Layered Halide Hybrid Perovskite Semiconductors," *ACS Nano*, vol. 10, pp. 9776-9786, 2016.

[48] K. Galkowski, A. Mitioglu, A. Miyata et al., "Determination of the exciton binding energy and effective masses for methylammonium and formamidinium lead tri-halide perovskite semiconductors," *Energy Environ. Sci.*, vol. 9, pp. 962-970, 2016.

[49] F. Ruf, M. F. Aygüler, N. Giesbrecht et al., "Temperature-dependent studies of exciton binding energy and phase-transition suppression in (Cs,FA,MA)Pb(I,Br)3 perovskites," *APL Mater.*, vol. 7, pp. 031113, 2019.

[50] H. T. J.-C. Blancon, W. Nie et al., "Extremely efficient internal exciton dissociation through edge states in layered 2D perovskites," *Science*, vol. 355, pp. 1288–1292, 2017.

[51] A. H. Proppe, R. Quintero-Bermudez, H. Tan et al., "Synthetic Control over Quantum Well Width Distribution and Carrier Migration in Low-Dimensional Perovskite Photovoltaics," *J. Am. Chem. Soc.*, vol. 140, pp. 2890-2896, 2018.

[52] N. Wang, L. Cheng, R. Ge et al., "Perovskite light-emitting diodes based on solution-processed self-organized multiple quantum wells," *Nat. Photonics*, vol. 10, pp. 699-704, 2016.

[53] M. B. Price, J. Butkus, T. C. Jellicoe et al., "Hot-carrier cooling and photoinduced refractive index changes in organic-inorganic lead halide perovskites," *Nat. Commun.*, vol. 6, pp. 8420, 2015.

[54] H. Bhatia, J. A. Steele, C. Martin et al., "Single-Step Synthesis of Dual Phase Bright Blue-Green Emitting Lead Halide Perovskite Nanocrystal Thin Films," *Chem. Mater.*, vol. 31, pp. 6824-6832, 2019.

[55] H. J. Julian A. Steele1, Iurii Dovgaliuk et al., "Thermal unequilibrium of strained black CsPbI_3_ thin films," *Science*, vol. 365, pp. 679–684, 2019.

[56] T. Chiba, Y. Hayashi, H. Ebe et al., "Anion-exchange red perovskite quantum dots with ammonium iodine salts for highly efficient light-emitting devices," *Nat. Photonics*, vol. 12, pp. 681-687, 2018.

[57] Y.-H. Kim, S. Kim, A. Kakekhani et al., "Comprehensive defect suppression in perovskite nanocrystals for high-efficiency light-emitting diodes," *Nature Photon.*, vol.15, pp. 2021.

[58] H. Amenitsch, M. Rappolt, M. Kriechbaum et al., "First performance assessment of the small-angle X-ray scattering beamline at ELETTRA," *J. Synchrotron Radiat.*, vol. 5, pp. 506-508, 1998.

[59] M. Burian, M. Porer, J. R. L. Mardegan et al., "Structural involvement in the melting of the charge density wave in 1T−TiSe2," *Phys. Rev. Res.*, vol. 3, pp. 013128, 2021.

[60] S. Ran, X. Zong, D. Fang et al., "Novel image analysis of two-dimensional X-ray fiber diffraction patterns: example of a polypropylene fiber drawing study," *J. Appl. Crystallogr.*, vol. 33, pp. 1031-1036, 2000.
